# Quantum Entanglement and State-Transference in Fenna–Matthews–Olson Complexes: A Post-Experimental Simulation Analysis in the Computational Biology Domain

**DOI:** 10.3390/ijms241310862

**Published:** 2023-06-29

**Authors:** Francisco Delgado, Marco Enríquez

**Affiliations:** 1 School of Engineering and Sciences, Tecnologico de Monterrey, Atizapan 52926, Mexico; 2 School of Engineering and Sciences, Tecnologico de Monterrey, Santa Fe 01389, Mexico

**Keywords:** Fenna–Matthews–Olson complex, light-harvesting complexes, quantum open systems, quantum entanglement, quantum state transfer, quantum coherence

## Abstract

Fenna-Mathews-Olson complexes participate in the photosynthetic process of Sulfur Green Bacteria. These biological subsystems exhibit quantum features which possibly are responsible for their high efficiency; the latter may comprise multipartite entanglement and the apparent tunnelling of the initial quantum state. At first, to study these aspects, a multidisciplinary approach including experimental biology, spectroscopy, physics, and math modelling is required. Then, a global computer modelling analysis is achieved in the computational biology domain. The current work implements the Hierarchical Equations of Motion to numerically solve the open quantum system problem regarding this complex. The time-evolved states obtained with this method are then analysed under several measures of entanglement, some of them already proposed in the literature. However, for the first time, the maximum overlap with respect to the closest separable state is employed. This authentic multipartite entanglement measure provides information on the correlations, not only based on the system bipartitions as in the usual analysis. Our study has led us to note a different view of FMO multipartite entanglement as tiny contributions to the global entanglement suggested by other more basic measurements. Additionally, in another related trend, the initial state, considered as a Förster Resonance Energy Transfer, is tracked using a novel approach, considering how it could be followed under the fidelity measure on all possible permutations of the FMO subsystems through its dynamical evolution by observing the tunnelling in the most probable locations. Both analyses demanded significant computational work, making for a clear example of the complexity required in computational biology.

## 1. Introduction

Photosynthesis is a key process intended to obtain energy from sunlight, and is vital for the survival of life on Earth. A series of physical and metabolic reactions occur in specialized structures to absorb, transfer, and store light energy efficiently within photosynthetic organisms. The importance of micro-organisms in anthropic activities has been highlighted nowadays as a third generation of biofuels, particularly those coming from photosynthetic ones [1], especially cyanobacteria [2], which are the main target of the current study due to the important role played by their dependence on temperature [3]. Among those structures, Light-Harvesting Complexes (LHC) are responsible for light capture and energy transference into the Reaction Centres (RC) [4], where electron transference takes place following the photosynthetic process. Due to their almost 100% efficiency, LHCs have been the subject of study, particularly in the quantum realm [5,6,7,8].

The current studies on LHCs cannot proceed without meeting of several necessary technological developments and achieving collaboration among different disciplines. An array of proteins in which the chlorophyll molecules are embedded to transfer light energy in the further processes within photosynthesis, LHCs appear to perform tasks concerning the most basic rules of matter, where our measurement instruments are not yet completely able to reach in understanding such mechanisms more deeply. Thus, a multidisciplinary approach is necessary to combine experimental, theoretical, and computational approaches. Such combined efforts have allowed us to unravel the mechanism behind the aforementioned energy transfer. Photonics has provided the basis for advanced femtosecond laser techniques to trace the excitation energy transfer (EET) dynamics [9,10], while biochemistry and genetics have contributed to the methods used to purify, characterize, and modify the protein structures necessary in spectroscopy essays [11,12,13,14,15,16]. While theoretical methods and computational models allow us to simulate the light absorption behaviour in different structural features [17], this is only affordable through computational biology, which provides observable data to compare with experimental mesoscopic observations. In fact, the computer atomistic modelling of LHC comprises multiscale methods involving dynamical and spectroscopic properties regarding excitation and site energies, excitonic couplings, and spectral densities determining the exciton dynamics and spectroscopic properties in the key aspects of photosynthesis [18].

Accordingly, LHCs are the focus of a multidisciplinary effort to understand their global behaviour. The growing interest in these structures is centred on their biological study, potential photovoltaic applications, and the natural structures that control certain quantum computing processes at room temperature [19,20]. All those processes lie in the common frontier of biology, chemistry, and physics. Biophysics, a discipline requesting all necessary physical theories and methods to understand how a biological system works, remains open to the most elusive phenomena behind life. There, the role of computational biology completes the analysis coming from the experimental data and further theoretical analysis.

Particularly, the Fenna–Matthews–Olson (FMO) complex is a photosynthetic trimer antenna present in anoxygenic photobacteria as the Green Sulfur Bacteria (GSB) [8]. In each monomer, it holds eight Bacteriochlorophylls-*a* photopigments (BChls-*a*) inside a protein scaffolding structure. These antennas capture photons, transferring light energy from FMO to RC through their eight BChls. The absorption and transfer mechanisms are not yet completely understood [21]. While thermal excitation in the complete structure alone could possibly be sufficient to understand this basic phenomenon and its efficiency, other analyses suggest the crucial intervention of quantum features to explain it. Thus, quantum biology is a biophysics subdiscipline accounting for the importance of quantum mechanical effects in living systems. FMOs are sufficiently simple life systems to understand the quantum behaviour involved within protein structures. Because certain topics involving the current research, particularly those related to quantum mechanics, could become difficult for inexperienced readers, we provide more friendly descriptions of the meaning of several concepts, phenomena, and equations throughout the presentation.

There exists an interesting series of experimental outcomes leading to a deeper understanding. A water-soluble pigment–protein complex was the first to be analysed by X-Ray spectroscopy [22,23]. In addition to its chemical structure, the identification of several photopigments [24] (the BChls) was attained by electron microscopy, which provided detailed and exact knowledge of their structure [5], distances, and orientations as well as their protein supporting structure. Such knowledge led to the theoretical settlement of the dipole–dipole interactions among BChls and precise quantification of the involved Hamiltonian [9,25] (without considering other secondary time-dependent interactions). This allowed the possibility of inquiring about other more subtle features of this structure using computer simulations [26]. Thus, compared with other observational entities, certain key constants rule their precise behaviour [27]. Through the corresponding computational analysis, at least for quantum purposes, several models analysing their dynamics have been implemented to reach more precise predictions fitting the facts revealed by spectroscopy [28,29]. Detailed research efforts concerning the FMO complex knowledge and their understanding can be found in [30].

**Figure 1:**
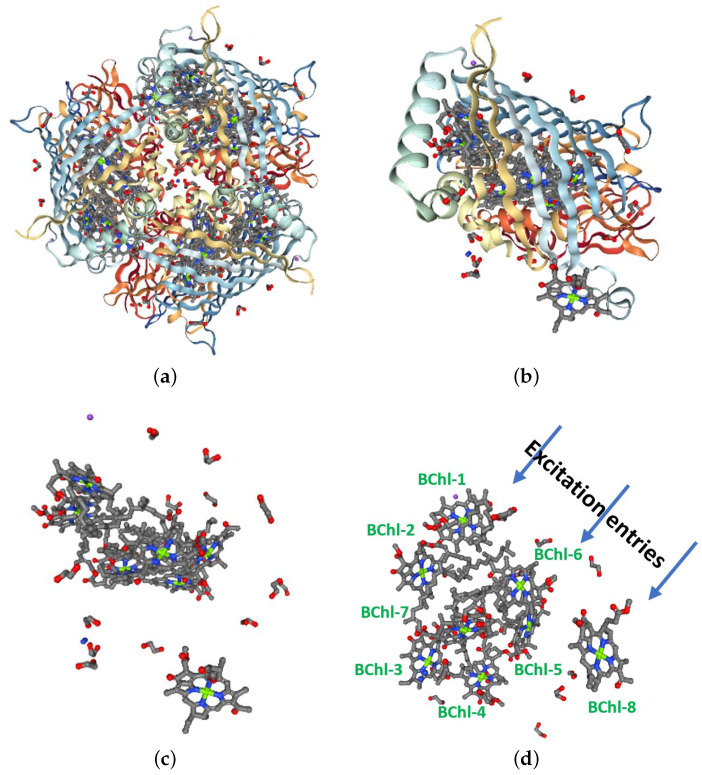
(**a**) FMO complex and its trimer structure, (**b**) FMO monomer, (**c**) monomer structure without its protein scaffolding, and (**d**) rotated monomer with the typical BChls numbering. Figure produced from Protein Data Bank file 3EOJ [31].

Figure 1 shows several views of the FMO complex [31]: (a) a whole view exhibiting its trimer structure, (b) one of the monomers with its protein scaffolding, (c) the same monomer free of its scaffolding, and (d) a rotation of the last monomer, showing the eight BChls in their commonest numbering. The interactions among the BChls are mainly dipolar electric. Note that each BChls has a specific orientation in terms of its dipolar momentum and that the eighth BChl is located inside the monomers (closer to the chlorosome baseplate, the region where the light energy comes in); for this reason, it was the last to be discovered and initial analyses did not consider it. In fact, BChls 3 and 4 are the nearest to the cytoplasmic membrane and RC. On the other hand, BChl 1, BChl 6, and BChl 8 are the closest to the chlorosome baseplate, where the light energy enters into FMO [5,8,32,33]. In fact, the BChl positions and orientations are interestingly associated with their own excitation energy range and their order in the energy transfer path, as observed in spectroscopic studies. Thus, these BChls are mainly responsible for the absorption energy face (the outer antenna towards blue), while those delivering the energy gathering and linked to the RC are shifted towards red [5]. The FMO complex is a photosynthetic component in all species of GSB. Of these species, *Chlorobaculum tepidum* [34] and *Prosthecochloris aestuarii* are the most deeply studied, exhibiting slight structural differences among certain strains belonging to each species.

The FMO became the first LHC structure to be mapped under spectroscopy [22,23]. The spectroscopic analysis demonstrated the existence of long-lived quantum coherence inside the FMO complex [35,36,37] together with its role and importance for its high energy transfer efficiency. Nevertheless, it is a matter of debate [38,39] between coherent quantum (e.g., Lindblad equation) and incoherent non-quantum (e.g., Förster theory) approaches to explain the efficient light-harvesting process useful for photosynthesis as well as their different timescales, questioning the relevance of quantum coherence in the process [38,40,41]. On the other hand, several studies have stated that FMO requires both coherent and incoherent energy transfers [36,42,43,44]. Together, theoretical physics and chemistry are developing a better understanding of the mechanisms involved under a quantum perspective by enabling computer simulations in the light-harvesting tracking [35,45,46].

In a simplified interpretation of the phenomenon, BChls interact among them through electrical forces. However, their protein scaffolding structure can be understood as springs between each pair of BChls exerting elastic forces. Thus, light excitation is transmitted through the entire elastic structure. Nevertheless, in the beginning, such transmission has a quantum nature, leaving room for slower classical and mechanical transmission [30]. Thus, the faster quantum mechanical excitation is ruled by features such as state superposition, coherence, and entanglement, which are explained below.

Quantum entanglement constitutes an important feature behind protein structures through the quantum coherence exhibited in the FMO. This phenomenon appears to be responsible for the set of correlated BChl behaviours that jointly transfer the light energy excitation under a process emulating the well-known quantum tunnelling effect. A clearer and more intuitive depiction of such phenomena is properly discussed in the development. This tunnelling effect and its understanding could become useful in the comprehension of the FMO as a quantum channel inside a biological system, as it has been tracked by spectroscopic methods [47]. However, entanglement is an elusive quantum property under the absence of a final absolute measure to comprise its richness and complexity. To inquire about such phenomena, condensed-matter models, including quantum mechanics, should be implemented by proper computer simulations considering their complex structure as a part of their protein scaffolding. Thus, mathematical modelling boosted by experimental observations in spectroscopy on biological samples has allowed post-experimental analyses to simulate those quantum features. In particular, and beyond the biological interest in these simple systems, both quantum entanglement and state transference through each monomer structure are interesting features in quantum information and processing.

The aim of the current research has two main directions inside the computational biology domain, sharing the frontier with quantum biology, first through the analysis of more complex entanglement models that are commonly recurred in the contemporary literature, and second to provide a certain insight into the state transfer through the excitation of light energy crossing the complex structure of the surrounding protein scaffolding. Section 2.1 synthetically presents the basic details of the quantum mechanical excitation modelling through each monomer inside the FMO. Such modelling allows us to simulate the time evolution of the excitation through a non-Markovian method. The obtained outcomes are the building blocks of the following sections. Section 2.3 contains a short review of entanglement measures commonly used or suggested in the study of FMO entanglement. We present a precise timely description of them along with their theoretical relations, followed by an analysis of specific examples with their similarities and discrepancies. Despite most of these measures referring to bipartition-based models, which involve the overall set of BChls, we introduce the maximum overlap with respect to the closest separable state, a foreseeable and promising quantum measure more appropriate for this phenomenon. Nevertheless, it requires considerable computing effort. We obtain the entanglement analysis of several important groups of BChls as a function of time and temperature. The fourth section proposes a model to track the state transference over time as departing from the initial condition stated in the second section to reach the tunnelling effect in the excitation. The final section concludes the main findings.

## 2. Results and Discussion

### 2.1. Fundamentals of FMO Dynamics Simulation Using the Hierarchical Equations of Motion

In this section, a brief review concerning the theoretical aspects relevant to modelling the excitation evolution inside the FMO is discussed, as well as other complementary references regarding each fundamental topic. Thus, we first discuss the main considerations about excitation among BChls together with their scaffold structure, then the quantum equations in the open quantum systems realm, where this problem should be analysed, in addition to their computational addressing. With these elements, it is possible to model the excitation evolution under specific initial conditions of excitation, which are be discussed as well. Finally, traditional measures of entanglement inside the group of BChls of each monomer are briefly studied; we improve upon such models in the next section.

**Figure 2:**
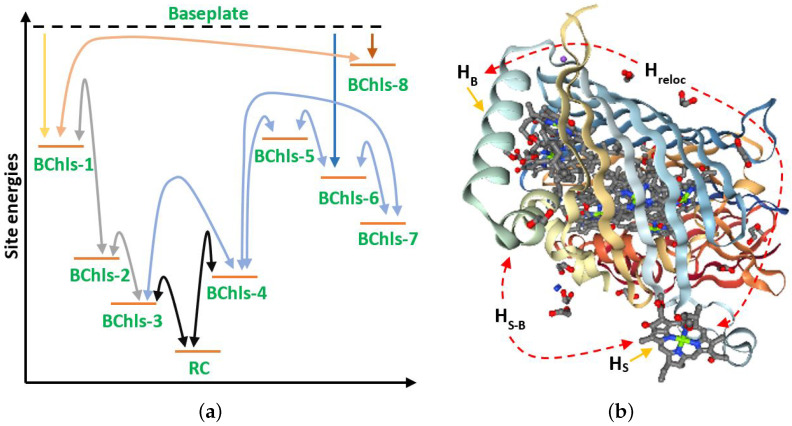
(**a**) Site energies transition series as observed by spectroscopy in FMO complex, noting the main energy entries through BChls 1, 8, and 6; and (**b**) main Hamiltonian terms: $H_{S}$, the oscillatory excitation of each BChl; $H_{\mathrm{reloc}}$, the BChl relocalization energy to obtain a BChl equilibrium position within the protein scaffolding (Bath); $H_{B}$, the energy of the protein scaffolding (Bath); and $H_{S}$, the interaction energy between each BChl (System) and the scaffolding (Bath). Figure produced from Protein Data Bank file 3EOJ [31].

When energy transmission through the FMO is settled, the excitation as vibrational modes through the BChls has a mainly quantum nature. This implies that several modes between each pair coexist at the same time in superposition, a feature present exclusively in quantum systems. This generates the energy levels and characteristic transitions depicted in Figure 2a regarding the excitation of each BChl, which means an increase and decrease from/to the ground energy level of each one. The phenomenon can be observed in the experimental spectroscopy analysis depicted below.

#### 2.1.1. FMO Structural Model

The FMO complex is a nanoscale water-soluble protein. It has a molecular weight of 150,000 Da, a maximum diameter of 8.3nm [22,23], and an average distance between nearest-neighbour BChls of 12 Å [13]. The proven approach to model the excitation evolution of BChls inside FMO first considers the group of eight BChls as the core quantum system [30]. In experimental spectroscopy analysis, it has been observed that each BChl exhibits a two-level system behaviour where in the main only one BChl is excited at a time, with bi-excitonic or higher states being rare [48]; this effect is due to and reinforced by the dipole blockade behaviour [49]. Thus, a preferred basis on which to depict the evolution is the occupation basis $\left|k\rangle \equiv \;\right|0_{1}...0_{k-1}1_{k}0_{k-2}...0_{8}\rangle$; alternatively, the excitonic basis $|\epsilon_{i}\rangle ,i=1,...,8$ corresponds to the free Hamiltonian eigenvalues of the system formed by the eight BChls [40].

As the knowledge of precise energies inside and together with the protein structure becomes a theoretical challenge, it is commonly obtained by fitting the optical spectra measured with spectroscopic techniques considering a femtoseconds range [9]. The Hamiltonians are reported in the following subsections and in Appendix A. In addition, the model should consider the interaction with the protein scaffolding. Despite its structural complexity, it can be considered as a continuous media stating a thermal bath, thereby moving the problem into an open system one. Such systems include the bath environment through quantum master equations (Lindblad [28,45] or Redfield [26,28]). More accurate approximations are reached using non-Markovian approaches such as the Hierarchical Equations of Motion (HEOM) [27,50,51]. Finally, to make the simulation implementation easier for the computer systems evolution, the superoperator method converts such problems into a finite set of first-order differential linear equations [30]. We briefly deal with such approaches in the next subsections.

#### 2.1.2. Interactions inside of FMO among Their Structural Components

The Hamiltonians for each monomer in the FMO are obtained by combining spectroscopy measurements and theoretical models. Figure 2a comprises several of the observed series of excitation dynamics. Main energy entries occur in the nearest BChls to the baseplate (1, 6, and 8) [5]. A cascade energy transition begins with the privileged paths shown there. In the more recent studies, BChl 8 appears as the main entry, despite it not being considered in the original analysis (where BChl 1 appeared as the main) because it was discovered later [23,31]. The final energy transfer moves on the RC through BChls 3 and 4 [51].

The full modelling for the interactions in each monomer considers the dipole–dipole interaction among BChls [28]. In spite of this, the structure becomes modified by the protein scaffolding stating new equilibrium positions [30]. Such a development introduces a relocalization energy $H_{\mathrm{reloc}}$ and a set of oscillatory energies around those new equilibrium points $H_{S}$ for the system of the set of eight BChls. This Hamiltonian has been reported for *C. tepidum* [24] and *P. aestuarii* [5]. We include such $H_{S}$ Hamiltonians in Appendix A. In addition, protein scaffolding (considered as a thermal Bath) has its own energy $H_{B}$ depending on several external parameters. Finally, other main terms consider the interaction between the System (the set of eight BCHls) and the Bath. Thus, the main Hamiltonian for the entire problem becomes $H=H_{S}+H_{\mathrm{reloc}}+H_{B}+H_{S-B}$. Figure 2b depicts graphically the last set of interactions on the monomer $H_{S}$ for the group of BChls $H_{\mathrm{reloc}}$ for the relocalization energy due to De protein folding, $H_{B}$ for the state of the phononic bath, and $H_{S-B}$ for the interaction between the main system and the bath.

#### 2.1.3. Lindblad and Redfield Equations for an FMO Monomer

We are interested in the joint behaviour of BChls, which is depicted as a statistical mixture of states through the density matrix written in terms of whether the occupation or the excitonic bases. Clearly, a first approximation is the Liouville–von Neumann equation:

(1) $$\begin{array}{c} i\hbar \frac{\partial \rho }{\partial t}=\left[H_{S},\rho \right] \end{array}$$

The time dependence of the set of BChls imposes a natural statistical mixture of quantum states. Nevertheless, $\rho =\rho_{S}$, and hence $H=H_{S}$, is unable to capture the interaction with the protein scaffolding, which is a complex and dense surrounding structure, which suggests treatment as a thermal bath. Otherwise, if ρ represents the entire system, it is not possible to isolate $\rho_{S}$, as in the further time-evolution both the BChls system and the bath become entangled regardless of the initial condition $\rho =\rho_{S}⊗\rho_{B}$. Concerning our interest in $\rho_{S}$ and the complexity of such a scaffolding, a better approximation is provided by the open quantum system approach, where the protein scaffolding is modelled as a thermal bath. Thus, the corresponding master equations provide a reasonable approximation. For instance, the Lindblad master equation [52]:

(2) $${\dot{\rho }}_{S}=-\frac{i}{\hbar }\left[H_{S},\rho_{S}\right]+\frac{1}{\hbar^{2}}\sum_{\alpha }\left(L_{\alpha }\rho_{S}L_{\alpha }^{†}-\frac{1}{2}\left\{\rho_{S},L_{\alpha }^{†}L_{\alpha }\right\}\right)$$

where $L_{\alpha }$, the so-called Lindblad operators, should integrate the interaction between the system and the bath. Despite this, such operators do not have a direct expression with the physical arrangement, instead forming a generic basis for the bath. Nevertheless, it has been used as an approximation of the FMO dynamics [43,45,53]. Alternatively, the Redfield master equation [54]:

(3) $${\dot{\tilde{\rho }}}_{S}=-\frac{i}{\hbar }\left[H_{S},\rho_{S}\right]-\frac{1}{\hbar^{2}}\sum_{\alpha }\left[\Sigma_{\alpha },\Lambda_{\alpha }\rho_{S}-\rho_{S}\Lambda_{\alpha }^{†}\right]$$

with ${\tilde{\Sigma }}_{i},{\tilde{\Lambda }}_{i}$ being operators which physically depict the bath coupling. This approach has been extensively used to model FMO behaviour [26,28,29].

In both cases, the later master equations are constructed to depict the main system (the set of BChls in each monomer) considering external interactions with the phononic bath (the protein scaffolding). While the Lindblad master equation is mainly constructed on a mathematical basis depicting the external interaction, the Redfield one involves a clearer and closer relationship with the involved physical systems. Nevertheless, there is criticism of such approaches concerning their Markovian nature. In fact, it is believed that the FMO dynamics depend not only on its present state but also on the past states, exhibiting a non-Markovian nature [55]. We deal with such a requirement in the following subsection.

#### 2.1.4. Hierarchical Equation of Motion Model and the Superoperator Approach of Master Equations

The Hierarchical Equation of Motion (HEOM) [50] is a non-Markovian model based on a non-perturbative approach. It was developed to reproduce the evolution of ρ for quantum dissipative systems such as FMO. After the following technical presentation, we discuss a more friendly interpretation of the HEOM method applied to our current problem. HEOM is constructed as a recursive set of equations considering *D* previous temporal stages as a bath memory. Each ρ stage is labelled with a vector n of dimension *N* (in this case N=8, the total number of BCHls). Then, $\rho_{n}$ with n=(0,..,0) corresponds to the real density matrix of the BChls system $\rho_{S}$. Other $\rho_{n}$ are auxiliary matrices regarding the entire global system memory. This set of matrices $\rho_{n}$ has a degree s=0,1,...,D related to all vectors $n=(n_{1},n_{2},...,n_{N})$, where ${0\leq n}_{k}\in Z^{+}\cup \left\{0\right\}$ with the property $\sum_{k=1}^{N}n_{k}=s$.

HEOM comprises the main features of the Markovian models as Redfield or Lindblad quantum master equations by including the Hamiltonian $H_{S}$ and the relocalization energy correction $H_{\mathrm{reloc}}$. In addition, the trapping from BChls 3 and 4 observed in the spectroscopy analysis, together with terms comprising the non-Markovian features, read as follows [30,50]:

(4) $$\begin{array}{ccc} \dot{\rho_{n}} & = & -\frac{i}{\hbar }[H_{S}+\sum_{k=1}^{N}\lambda_{k}|k\rangle \langle k|,\rho_{n}]-\sum_{k=1}^{N}n_{k}\gamma_{k}\rho_{n}-r_{\mathrm{trap}}\left(\{|3\rangle \langle 3|,\rho_{n}\}+\{|4\rangle \langle 4|,\rho_{n}\}\right)\\ & & \;+i\sum_{k=1}^{N}\Delta_{k}\left(\left[V_{k},\rho_{n_{k+}}\right]+n_{k}\left(\left[V_{k},\rho_{n_{k-}}\right]-i\frac{\beta \hbar \gamma_{k}}{2}\left\{V_{k},\rho_{n_{k-}}\right\}\right)\right) \end{array}$$

in the above expression, *k* is the Boltzman constant and β=kT; moreover, $V_{k}=\left|k\rangle \langle k\right|$, $\Delta_{k}=\lambda_{k}/\beta \hbar^{2}\gamma_{k}$ ($\lambda_{k}$ is the relocalization coefficient for k−th BChl [30]), and $\gamma_{k}$ can be considered as the bath cutoff frequency characterizing its non-Markovian nature [56]. Thus, $\gamma_{k}$ is the interaction strength between the bath and the k−th BChl; because BChls 3 and 4 drive the energy on the RC with a trapping rate $r_{\mathrm{trap}}$, it is represented in (4). There, if n is a vector of order *s*, then $n_{k\pm }$ is a vector of order s±1 identical to n but with the component $n_{k}$ increased (decreased) by one. Together, $\rho_{n}\left(0\right)$ for n≠0 are initially settled as the zero matrix. Finally, $n_{k+}=0$ is selected as the cut-off for n of order *D*.

Initial conditions (t=0) for the FMO system correspond to $\rho_{S}$ unentangled with the bath. Because it has been observed in spectroscopic studies that BChls 1, 6, and 8 are the antennas of FMO, $\rho_{S}\left(0\right)=\rho_{\mathrm{LOC}}^{k}=\left|k\rangle \langle k\right|$ with k=1,6,8 is widely considered as an initial condition in the literature [5,51,57]. Despite this, another kind of initial condition corresponds to the Förster Resonance Energy Transfer (FRET) [41], a feasible initial condition related to the fluorescence, a mechanism of energy transfer between light-sensitive molecules as the BChls. This suggests energy transference from a main BChl into the entire system as a function of their proper energies [42]:

(5) $$\begin{array}{c} \rho_{\mathrm{FRET}}^{i}=\sum_{k=1}^{N}|\langle \epsilon_{k}|i\rangle {|}^{2}|\epsilon_{k}\rangle \langle \epsilon_{k}|,\;i=1,...,8 \end{array}$$

where $\left\{|\epsilon_{k}\rangle |k=1,...,N\right\}$ are, as explained before, the eigenvalues of $H_{S}$. Commonly, i=8 is used as the main entry of light energy into each monomer of FMO.

**Figure 3:**
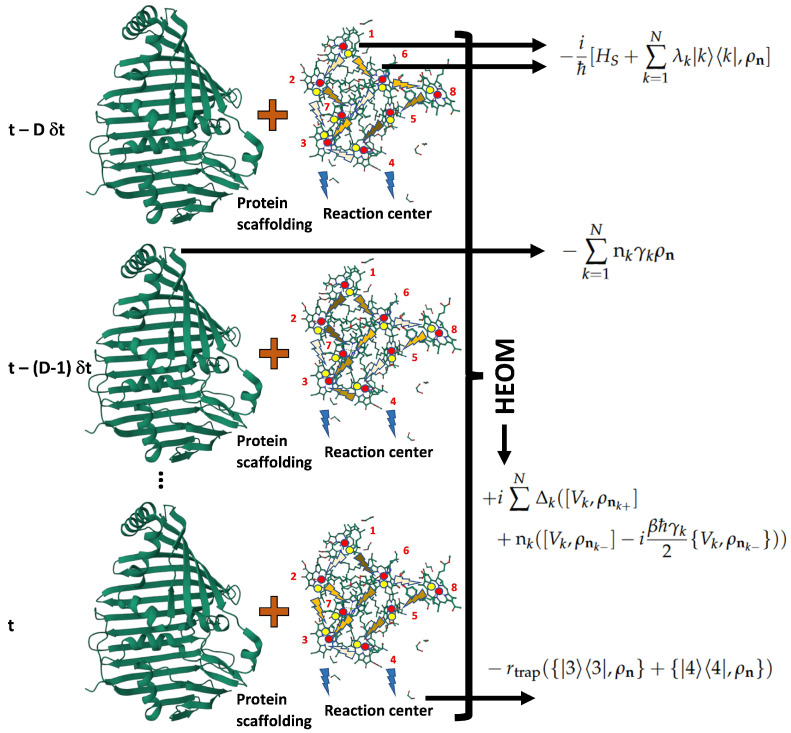
A series of partial states determining the behaviour of a further future state through the HEOM approach. Several terms in the master equations are related to physical elements in the FMO: dipole–dipole electric interactions among the BCHls and the relocalization of BChls due to their interaction with the protein scaffolding, the phononic bath contribution, the iterative HEOM approach connecting the partial states, and the trapping term providing the entrapment of excitation on BChls 3 and 4 (from above to below). Figure produced from Protein Data Bank file 3EOJ [31].

Clearly, the HEOM approach has a more complex interpretation due to its non-Markovian basis; it is pictorially summarized in Figure 3. Through several D+1 time steps, the complete system (with the BChls represented with the ball and stick models and the bath as protein alpha-helices and beta-sheets in green) evolves ruled by the electric interaction $H_{S}$ between BChls, in agreement with a theoretical analysis bas on the experimental spectroscopy observation and their mutual interaction. Interactions are shown with rays in different colours representing the quantum excitation modes. This representation can be useful in understanding the entanglement concept in the model. In the upper formula in Figure 3, in a first approximation [30], this interaction shifts the positions of the BChls, providing a relocalization energy $H_{\mathrm{reloc}}=\sum_{k=1}^{N}\lambda_{k}\left|k\rangle \langle k\right|$ (the shifting of the electrostatic point of equilibrium from red dots to yellow ones). Such an energy reference can be compared with that generated by gravity on a vertical spring by shifting the equilibrium position. In our model, negative trapping energy is introduced in the form of $-r_{\mathrm{trap}}\{|3\rangle \langle 3|+|4\rangle \langle 4|,\rho_{n}\}$ (note that below this term already includes the non-Markovian HEOM approach), reflecting the final delivery to the RC (the last formula in the figure). In other approaches [57], the RC is modelled as an additional system in the form of a sink, reflecting the excitement from the BChls system. Additionally, the effect of bath energy on the main system is introduced by $H_{B}=-\sum_{k=1}^{N}n_{k}\gamma_{k}\rho_{n}$ (the second formula in the figure). Finally, the HEOM approach relates the quantum state in each time step through the Hamiltonian in the third formula to obtain a future quantum state through the derivative of $\rho_{n}$ on the left side in (4).

Because HEOM requires a numerical approach, as is generally the case in all master equations, a computational procedure is convenient. In fact, the HEOM and open quantum system master Equations (2)–(4) can be transformed into a set of differential linear equations. This is known as the superoperator-supervector procedure [30]. In this procedure, the density matrix is considered as a supervector with two indexes, while each pair of matrices multiplying it on each side is considered a tensor product of supervectors, thereby generating a superoperator:

(6) $${\left(A\rho B\right)}_{ij}=\sum_{k,l}A_{ik}\rho_{kl}B_{lj}=\sum_{k,l}{\left(A⊗B^{T}\right)}_{ij,kl}\rho_{kl}$$

This rule can be applied in each term of (2)–(4), thereby jumping ρ as a supervector of size $N^{2}=64$ on the right side of each term through other operators and becoming multiplied by a superoperator κ of size $N^{2}\times N^{2}$ obtained by summing the tensor products appearing in each master equation:

(7) $${\dot{\vec{\rho }}}_{S}=\kappa_{\mathrm{HEOM}}\;{\vec{\rho }}_{S}$$

Thus, such a single transformation allows us to change any quantum master equation into a linear single matrix differential equation. This procedure allows an easy computer algorithm to b implemented to obtain the evolution of $\rho_{S}$. This strategy is simply a method of translating the HEOM equations into a single linear differential problem which can be easily numerically solved. A more detailed implementation of this procedure and of the HEOM method is reported in [30].

#### 2.1.5. Complementary and Operative Information of the BChls Dynamics Simulation

Certain operative aspects should be stated before the performance of the primary simulation to reach the oscillation dynamics for the eight BChls. Because of the important role of spectroscopy in the FMO dynamics, the constant parameters involved, such as $r_{\mathrm{trap}},\lambda_{k}$, and $\gamma_{k}$, which are typically expressed in energy units, are expressed in spectroscopy units instead (see Section 3).

Following the last statement, the constant $r_{\mathrm{trap}}=1\;{\mathrm{ps}}^{-1}$ is responsible from the trap efficiency of the BChls 3 and 4 energy towards the end of the process. This value is approximated by spectroscopy observations. Note that in our model we omit the final entrapment by the RC (as an example, see the modelling by [57]); thus, it is expected that energy finally becomes gathered by this pair of BChls. Reorganization energies are ruled by $\lambda_{k}$. This constant reflects the architecture of BChls inside FMO, and is responsible for the decoherence strength. Despite differentiated values for each BChl being expected, our actual knowledge only allows us to estimate them in the range $\left[35,65\right]\;{\mathrm{cm}}^{-1}$ [58,59]. Both extremes are commonly analysed in the literature by taking each value for the overall BChls. Similarly, the bath cutoff frequency $\gamma_{k}$ (the inverse of the bath coherence time-scale) is experimentally approximated at around $50\;{\mathrm{cm}}^{-1}$ [56,59].

An external physical parameter is the operation temperature *T*. The entire behaviour of FMO dynamics is influenced by it, including equilibrium populations, quantum coherence time, and energy capture efficiency. Extreme values used to observe the FMO behaviour range from experimental spectroscopy analysis temperatures (77K) to hot water temperatures around 333–343 K (60–70 °C) near the thermal vents where bacteria survives in absence of sunlight [12], living from the dim glow at a depth of 2500m. For this reason, a range of [77,347]K is commonly considered of interest in the simulation analysis. The following HEOM simulations were performed for a depth D=4. Despite higher values for *D* having been performed and reported [27], our results remain in the percentual variation range of $10^{-4}$.

### 2.2. Quantum Dynamics Modelling of BChls Excitation in the FMO Complex

In this subsection, we graphically report the data which are the basis for our entanglement and state transfer analysis. Operative details are summarized in Section 3.

Using the HEOM method, the parameters $r_{\mathrm{trap}}=1\;{\mathrm{ps}}^{-1},\gamma_{k}=50\;{\mathrm{cm}}^{-1}$, and the pair of values $\lambda_{k}=35,65\;{\mathrm{cm}}^{-1}$ (the same values for each BChl, k=1,...,8), a set of computer simulations using D=4 between t∈[0,15]ps were performed for *P. aestuarii* and *C. tepidum*. A wide group of representative temperatures were used: T=77,131,185,239,293,347K. The use of $\rho \left(0\right)=\rho_{\mathrm{FRET}}^{8}$ as an initial condition was implemented in all simulations. Several of these outcomes have been presented (*P. aestuarii*) in a limited previous analysis of single bipartite entanglement and coherence [60,61]. This work widely extends their analysis by considering another bacteria specie, and particularly by doing so under a more formal and diverse analysis of entanglement.

**Figure 4:**
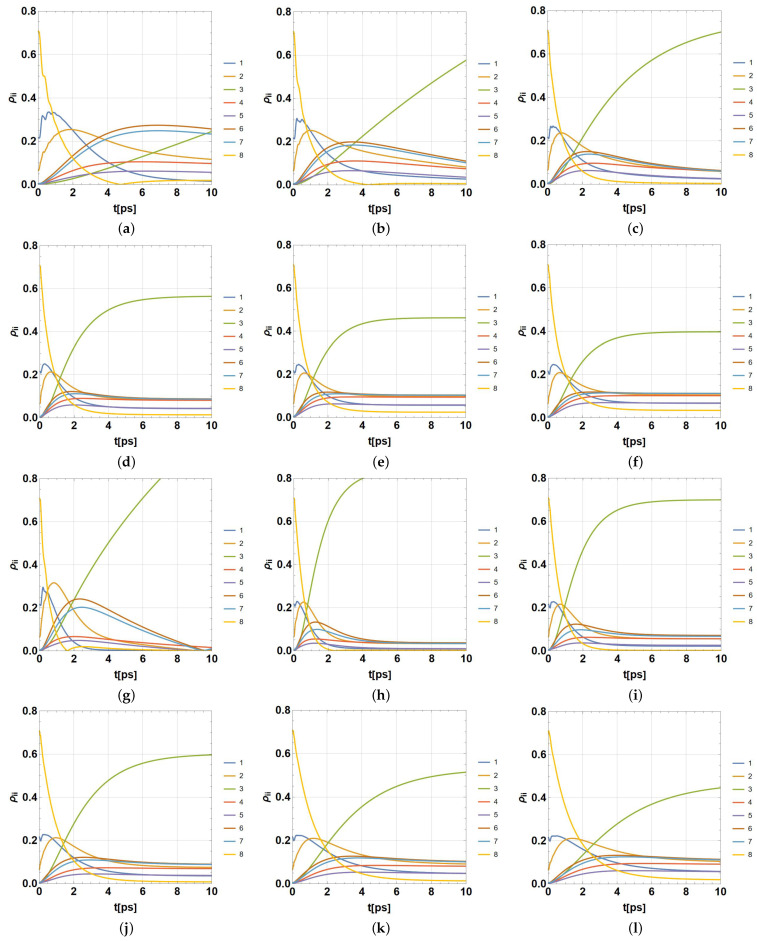
Population dynamics in [0,10]ps in color for each *i*-th BChl $\rho_{ii},i=1,...,8$ in *P. aestuarii*. (**a**–**f**) for $\lambda_{k}=35\;{\mathrm{cm}}^{-1}$ and (**g**–**l**) for $\lambda_{k}=65\;{\mathrm{cm}}^{-1}$, in both cases for ordered temperatures 77,131,185,239,293,347K, respectively, with $\gamma_{k}=50\;{\mathrm{cm}}^{-1}$ and $r_{\mathrm{trap}}=1\;{\mathrm{ps}}^{-1}$.

**Figure 5:**
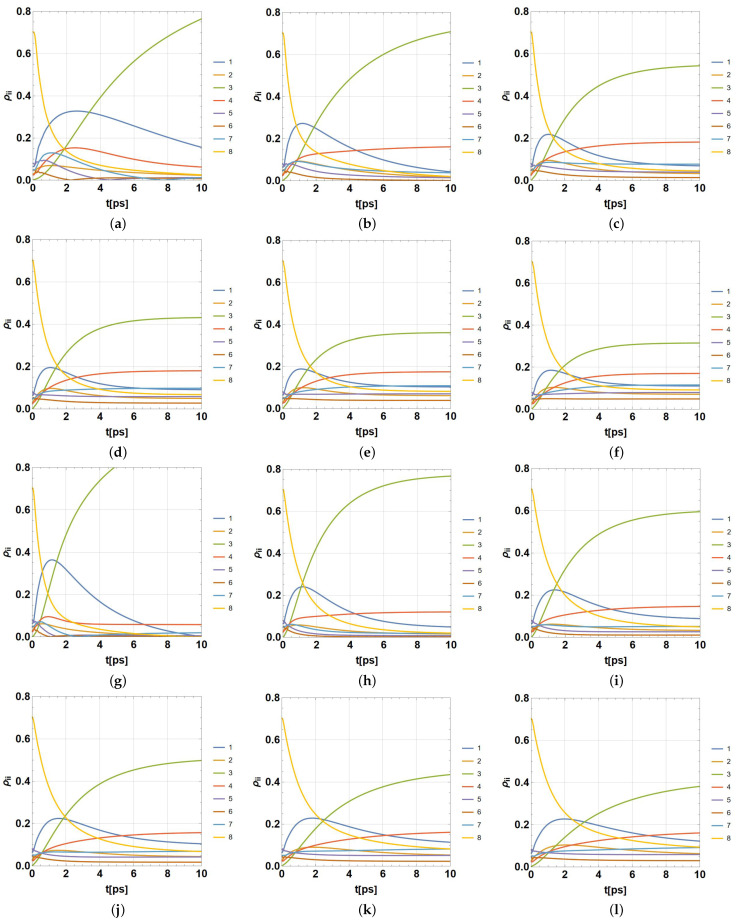
Population dynamics in [0,10]ps in color for each *i*-th BChl $\rho_{ii},i=1,...,8$ in *C. tepidum*. (**a**–**f**) for $\lambda_{k}=35\;{\mathrm{cm}}^{-1}$ and (**g**–**l**) for $\lambda_{k}=65\;{\mathrm{cm}}^{-1}$, in both cases for ordered temperatures 77,131,185,239,293,347K, respectively, with $\gamma_{k}=50\;{\mathrm{cm}}^{-1}$ and $r_{\mathrm{trap}}=1\;{\mathrm{ps}}^{-1}$.

The meaning of outcomes comprised in ρ(t) should be interpreted remembering that the evolution process produces a statistical mixture of quantum states, each one being a superposition of excitation modes between pairs of BCHls. Then, the diagonal entries of ρ(t), $\rho_{ii}\left(t\right),i=1,...,8$ represent the probability of each BChl becoming the excited one, considering that only one becomes excited at time *t*. Outcomes are plotted in Figure 4 (*P. aestuarii*) and Figure 5 (*C. tepidum*). In both cases, subfigures (a–f) correspond to $\lambda =35\;{\mathrm{cm}}^{-1}$ and subfigures (g–l) to $\lambda =65\;{\mathrm{cm}}^{-1}$. Only the populations $\rho_{ii},i=1,...,8$ are shown as functions of t∈[0,10]ps (the initial part of the simulated dynamics). In each group, the temperatures are ordered from lower to higher. The population for each BChl is presented in colour, in agreement with the colour legend on the right side. An important aspect not shown here (previously analysed [61]) is the increase in decoherence, which means the absence of quantum features through time. In fact, for higher times than 15ps the coherence (the diagonal-off entries in ρ(t)) drops to zero, reaching a classical statistical mixture of physical states (populations without quantum properties).

The effect of the balance on the final populations $\rho_{33}$ and $\rho_{44}$ is notable when the temperature rises. In addition, the reduction of coherence timelapse is notable with its increase (asymptotic behaviour of populations). The effect of $\lambda_{k}$ occurs in combination with *T*, noting that a higher $\lambda_{k}$ value accelerates the dynamics for the lower temperatures and slows down with the higher ones. We consider the entire data of those numerical simulations in order to inquire more deeply into the entanglement phenomenon among BCHls under several models of entanglement measures, as well as both semi-locally and globally. The development and outcomes are presented in the next section.

### 2.3. Entanglement and Quantum Coherence Measures

The eight BChls in each monomer of FMO evolved according to the HEOM stated in (4) and the initial condition $\rho_{\mathrm{FRET}}$ are considered. The states of such sets of BChls become mixed and entangled in general. Entanglement is a quantum feature that is not present in classical systems. While classical systems exhibit certain independence among physical dynamic variables or observables, such variables can only be weakly related through functional relations. Instead, certain groups of quantum observables can be strongly related in a superposition of such correlations. This means that upon measurement of just one of those observables, the remainder becomes strictly determined. Thia property belongs to the quantum state, not to the system itself. When no correlations are present among the related observables, we can say that the quantum state is separable.

Nevertheless, this apparent single feature becomes highly complex to quantify when the number of subsystems increases. For instance, for a pair of systems exhibiting two levels (with observables as non-excited or excited for each one, as BChls are), there is a single type of entanglement when considering both systems together. For the same case, three systems could exhibit correlations by pairs or for all three together. For eight BChls, each one excited or not, the type of correlations increases dramatically, by pairs, by thirds, etc., and then again through subgroups having correlations. Thus, the quantification of entanglement becomes highly complex, as there exists no clear measure comprising this richness.

Only scarce measures of bipartite pure systems are well known (those not involving statistical mixtures of quantum states), despite there being certain useful measures for a limited group of subsystems. For mixed states, the situation is more intricate around the quantification of entanglement, as there are both statistical and quantum correlations in the same system. The success of bipartite systems lies in their simplicity as well as in that of the Peres—Horodecki criterion [62,63] for a pair of subsystems. Clearly, such a criterion could be applied to larger systems by considering bipartitions, which means splitting the system into two parts, each one possibly containing more than a single system. Such a measure provides the correlation only between the subsystems, not inside each one.

For this reason, the most common measures considered to quantify entanglement in each monomer of the FMO are based on bipartitions. In this section, we pursue an analysis of the entanglement inside the FMO to characterize it as a function of parameters or their biological properties.

#### 2.3.1. Analysis under Several Measures of Global and Bipartite Entanglement

In [30], an introductory analysis was presented for models with N=7; however, a deeper analysis can be performed using improved entanglement measures. In the literature, the more immediate and recurrent measures correspond with the *concurrences* [26,30,60,64]:

(8) $$\begin{array}{ccc} C_{\left\{kl\right\}} & \equiv & C\left(\rho_{\left\{kl\right\}}\right)=2\left|\rho_{kl}\right| \end{array}$$

(9) $$\begin{array}{ccc} C_{\left\{k\right\}} & \equiv & C\left(\rho_{\left\{k\right\}}\right)=2\sqrt{\rho_{kk}\left(1-\rho_{kk}\right)} \end{array}$$

both obtained by partial tracing and using the criterion developed in [27,63]. These measures, the concurrences, are simply mathematical functions quantifying the bipartite entanglement in a specific order, i.e., zero for separable states and one for maximal entanglement, with tighter correlations. They are a kind of measure belonging to the monotones of entanglement, a wider group of ordered measures ranging from separability (minimum) to maximal entanglement (maximum).

In the previous formulas, $\rho_{\left\{A\right\}}$ is the tracing of ρ on the entire system, with the exception of those subsystems in the set *A*. Both measures report entanglement in the range [0,1], 0 for separability and 1 for maximal entanglement. For these reasons, sometimes the squares of (8) and (9) are considered as the concurrence definitions. In general, they provide certain local information about entanglement (between two BChls or between one and the rest, respectively); however, they remain limited. In particular, the local paired concurrence $C_{\left\{kl\right\}}$ becomes naturally related with the coherence [65] of the system (using the measure $l_{1}$-norm): $C_{l_{1}}\left(\rho \right)=\min_{\sigma \in \Gamma }\sum_{ij}|\rho_{ij}-\sigma_{ij}|=2\sum_{i<j}\left|\rho_{ij}\right|$ [66]. Quantum coherence is understood as the stage at which a quantum system exhibits a superposition of classical physical states; otherwise, the system exhibits decoherence. For a mixed state, it may be noted that when only a statistical mixture of states remains, as in our case, it means that ρ(t) becomes practically a diagonal matrix in the occupation basis (their diagonal-off entries are practically zero). In that sense, both $C_{\left\{kl\right\}}$ and $C_{l_{1}}\left(\rho \right)$ respectively function as local and global entanglement measurements [27].

Other partitioned or global entanglement measures have been implemented in the entanglement analysis of FMO. For instance, in [67], *logarithmic negativity* was employed by defining a bipartition k,N−k of BChls A|B: $\left\{{\mathrm{BChl}}_{i_{1}},...,{\mathrm{BChl}}_{i_{k}}\right\}\to A=\left\{i_{1},...,i_{k}\right\}$ and $\left\{{\mathrm{BChl}}_{i_{k+1}},...,{\mathrm{BChl}}_{i_{N}}\right\}\to B=\left\{i_{k+1},...,i_{N}\right\}$. The expression for the logarithmic negativity for this bipartition considering the single excitation states model becomes

(10) $$\begin{array}{ccc} E_{A|B}^{\mathrm{LN}}\left[\rho \right] & = & \log_{2}\left(1+2\sqrt{\sum_{i\in A}\sum_{j\in B}{\left|\rho_{ij}\right|}^{2}}\right)=\log_{2}\left(1+\sqrt{E_{A|B}^{N}\left[\rho \right]}\right) \end{array}$$

(11) $$\begin{array}{ccc} & & \mathrm{with}:\;E_{A|B}^{N}\left[\rho \right]\equiv \sum_{i\in A}\sum_{j\in B}C_{\left\{ij\right\}}^{2} \end{array}$$

which is a bipartitioned entanglement measurement. Note that the inner double sum on the partition elements inside the square root, $E_{A|B}^{N}\left[\rho \right]$, provides a cumulative entanglement measure on each partition, the *negativity.*

This measure clearly remains in the interval [0,Nk]. The sum over all partitions can provide a global measurement achieved with little computing effort (note that in the partitions for k=[N/2]=N/2 only half of them are different). In any case, this measure accounts for an additive amount of overall paired entanglement among all BChls. In other approaches, the *relative entropy* of entanglement has been used [68]:

(12) $$\begin{array}{ccc} E^{\mathrm{RE}}\left[\rho \right] & = & -\sum_{i=1}^{N}\log_{2}\rho_{ii}-S\left(\rho \right) \end{array}$$

(13) $$\begin{array}{ccc} & & \mathrm{with}:\;S\left(\rho \right)=-\mathrm{Tr}\left(\rho \log_{2}\rho \right) \end{array}$$

providing a global entanglement measure, which regards the information contained in the state. Another global measure is the Meyer–Wallach one for the system of BChls. It is a monotone measure [69] considered as an entanglement measure for several two-level systems together. For *N* BChls, the *Meyer–Wallach entanglement* measure is based on the entanglement of each BChls with the remainder, as follows:

(14) $$\begin{array}{ccc} E^{\mathrm{MW}}\left[\rho \right] & = & \frac{1}{N}\sum_{k=1}^{N}2\left(1-\mathrm{Tr}\left[\rho_{\left\{k\right\}}^{2}\right]\right)=\frac{1}{N}\sum_{k=1}^{N}4\rho_{kk}\left(1-\rho_{kk}\right)=\frac{1}{N}\sum_{k=1}^{N}C_{\left\{k\right\}}^{2} \end{array}$$

becoming the average quadratic concurrence $C_{\left\{k\right\}}^{2}$ of each BChl. Here, as depicted before, $\rho_{\left\{k\right\}}$ is the reduced density matrix for the *k*-th BChl obtained after tracing out all the remaining qubits [30,60]:

(15) $$\begin{array}{ccc} \rho_{\left\{k\right\}} & = & {\mathrm{Tr}}_{\left\{k\right\}}\left(\rho \right)=\left(1-\rho_{kk}\right)\left|0\rangle \langle 0\right|+\rho_{kk}\left|1\rangle \langle 1\right| \end{array}$$

In this interpretation, it can be visualized as an average measure of how each BChl remains entangled with its BChl partners. Nevertheless, this measure has inherent deficiencies, as it is unable to distinguish fully non-separable states from those with separable subsystems. Despite this, it is useful for obtaining a global vision of entanglement in the group of *N* BChls [70].

An alternative approach to provide a global measure of entanglement is a *weighted average of entanglement entropies* upon all bipartitioned groups of BCHls [70,71]:

(16) $$\begin{array}{ccc} E_{\tau }^{\mathrm{WAE}}\left[\rho \right] & = & \frac{1}{\left[N/2\right]}\sum_{m=1}^{\left[N/2\right]}\tau^{\left(m\right)}\left[\rho \right] \end{array}$$

(17) $$\begin{array}{ccc} \tau^{\left(m\right)}\left[\rho \right] & = & \frac{1}{N^{\left(m\right)}}\sum_{n=1}^{N^{\left(m\right)}}\tau \left(n\right)\left[\rho \right] \end{array}$$

Each *m*-bipartition splits the set of *N* BChls in two groups, for instance, $\left\{{\mathrm{BChl}}_{i_{1}},...,{\mathrm{BChl}}_{i_{m}}\right\}$ and $\left\{{\mathrm{BChl}}_{i_{m+1}},...,{\mathrm{BChl}}_{i_{N}}\right\}$. An m−bipartition has $N^{\left(m\right)}$ different elements, provided by Nm if m≠N/2 and 12Nm if m=N/2. In these expressions, [·] is the floor function. Summing the total number of bipartitions, we obtain $\sum_{m=1}^{\left[N/2\right]}N^{\left(m\right)}=2^{N-1}-1$. Despite τ(n)[ρ] being any bipartite entanglement measure, the *normalised negativity*τ(n)[ρ] [63] has commonly been used [70]:

(18) $$\begin{array}{ccc} \tau (n)[\rho ] & = & \frac{2}{2^{m}-1}\sum_{i}\left|\lambda_{i}\right| \end{array}$$

which is the normalised sum of the negative eigenvalues $\lambda_{i}$ of the partial transpose matrix associated with each bipartition. Despite being global, each weighted contribution ${E_{\tau }^{\mathrm{WAE}}}^{\beta }\left[\rho \right]={\left[N/2\right]}^{-1}{N^{\left(m\right)}}^{-1}\tau \left(n\right)$ can be considered as a specific entangled measure for the corresponding *m*-bipartition $\beta =i_{1},...,i_{m}|i_{m+1},...,i_{N}$. It is a more complex partitioned entanglement measure than (10), requiring a more powerful computer effort. Despite this, the main contribution of this measure is the weighted interpretation to reach an entanglement average; nevertheless, the exchangeable normalised negativity becomes more difficult to manage.

The last formulation can be alternatively applied using $E_{A|B}^{N}\left[\rho \right]$ instead of τ(n)[ρ], thereby defining $E_{E^{N}}^{\mathrm{WAE}}\left[\rho \right]$. While this measure uses the same set of bipartitions, it is a more economic measure in terms of computer calculations. In a further development, the complete set of bipartitions P is assumed in the following natural order:

(19) $$\begin{array}{ccc} P=\{P_{1},P_{2},P_{3},P_{4}\} & \equiv & \{\{\{1\},\{2\},...,\{8\}\},\\ & & \{\{1,2\},...,\{7,8\}\},\\ & & \{\{1,2,3\},...,\{6,7,8\}\},\\ & & \left\{\{1,2,3,4\},...,\{1,6,7,8\}\}\right\} \end{array}$$

In turn, each element of $P_{m}$ is labelled as $P_{m}^{n},n=1,...,N^{\left(m\right)}$. Then, the last construction, $E_{E^{N}}^{\mathrm{WAE}}\left[\rho \right]$, can be made regarding only the main part prescription $A=P_{m}^{n}$ of order m=1,2,...,[N/2]=4, as the remaining part *B* is easily obtained from each *A* as its complement $B=A^{C}$. Note that $P_{4}$ only contains the different bipartitions, one-half of the total. Thus, the number of elements in each order of bipartitions is 8,28,56,35 for k=1,...,4, respectively, for a total of 127 bipartitions. Regarding the entire presentation at this point, it is noticeable that the entanglement measures presented before are related to the simplest measures in (8) and (9) as an additive or averaged quantity in each case, based on bipartitions, mainly because of the Peres–Horodecki criterion.

**Figure 6:**
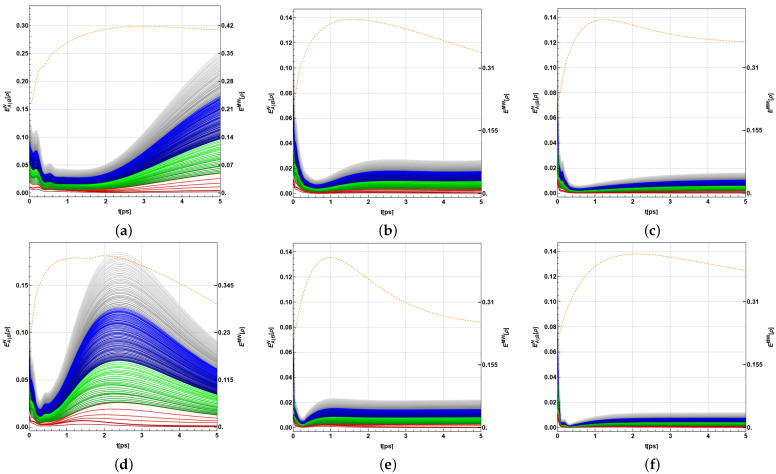
Comparison between $E_{A|B}^{N}\left[\rho \right]$ (additive bipartitions; k=1 red, k=2 green, k=3 blue, k=4 grey) read on the right scale and $E^{\mathrm{MW}}\left[\rho \right]$ (dashed orange line) read on the left scale. (**a**–**c**) for $\lambda_{k}=35\;{\mathrm{cm}}^{-1}$ and (**d**–**f**) for $\lambda_{k}=65\;{\mathrm{cm}}^{-1}$. Each set of three shows T=77,181,293K, respectively. In all cases, $\gamma_{k}=50\;{\mathrm{cm}}^{-1}$ for *P. aestuarii*.

**Figure 7:**
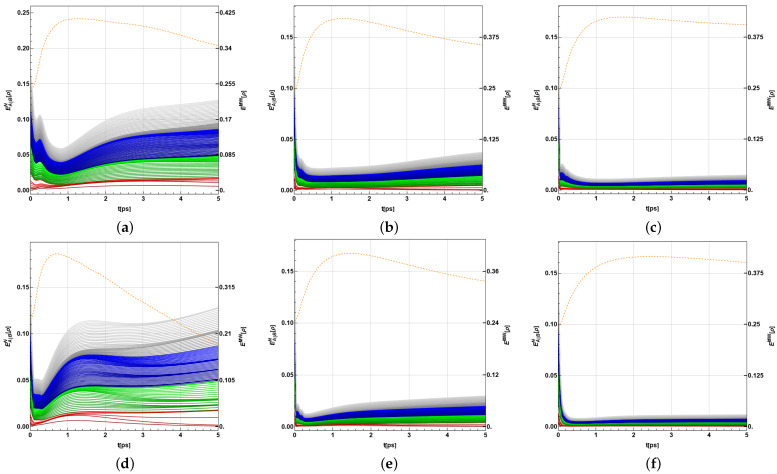
Comparison between $E_{A|B}^{N}\left[\rho \right]$ (additive bipartitions: k=1 red, k=2 green, k=3 blue, k=4 grey) read on the right scale and $E^{\mathrm{MW}}\left[\rho \right]$ (dashed orange line) read on the left scale. (**a**–**c**) for $\lambda_{k}=35\;{\mathrm{cm}}^{-1}$ and (**d**–**f**) for $\lambda_{k}=65\;{\mathrm{cm}}^{-1}$. Each set of three shows T=77,181,293K, respectively. In all cases, $\gamma_{k}=50\;{\mathrm{cm}}^{-1}$ for *C. tepidum*.

Thus, in the following analysis, we have initially compared $E_{A|B}^{N}\left[\rho \right]$ (averaged as in $E_{E^{N}}^{\mathrm{WAE}}\left[\rho \right]$) and $E^{\mathrm{MW}}\left[\rho \right]$ in Figure 6 (*P. aestuarii*) and Figure 7 (*C. tepidum*) in [0,5] ps, where the entanglement values become more important. Note that $E_{A|B}^{N}\left[\rho \right]$ is a direct summative entanglement property based on the entire possible bipartitions settled by P and measured by the paired squared concurrences $C_{\left\{kl\right\}}$ between single BChls belonging to each group of the bipartition. Thus, this cumulative quantity can be represented as piled to obtain a global entanglement measurement considering the entire set of bipartitions, as in these figures. This allows us to identify the contribution of each bipartition and each bipartition order at each time in the evolution. Moreover, the last piling is not only the simple sum of each $E_{A|B}^{N}\left[\rho \right]$; instead, each contribution is re-scaled as in $E_{\tau }^{\mathrm{WAE}}$, except more precisely, using $E_{A|B}^{N}\left[\rho \right]$ as τ(n). While, $E^{\mathrm{MW}}\left[\rho \right]$ is a global entanglement measure based on bipartitions, in this case it uses the single average of the entanglement squared concurrence of each BChl with the remaining system. Clearly, in the complexity of the entanglement measurement for multipartite systems, both measures provide different information about this feature; however, they both try to provide a global entanglement measurement.

Panels (a–c) show the case for $\lambda_{k}=35\;{\mathrm{cm}}^{-1}$ and (d–f) show the case for $\lambda_{k}=65\;{\mathrm{cm}}^{-1}$. Each set corresponds to T=77,181,293K. In all cases, $\gamma_{k}=50\;{\mathrm{cm}}^{-1}$. $E^{\mathrm{MW}}\left[\rho \right]$ is a global entropy measure obtained from the sum of entropies between each BChl and its remainder. It is shown by the dashed orange line, and should be read on the right scale. On the other hand, $E_{A|B}^{N}\left[\rho \right]$ is a bipartite measure between each bipartition in (19). It is shown (scaled as in the average of $E_{E^{N}}^{\mathrm{WAE}}\left[\rho \right]$) for each set of bipartitions with orders k=1 (red), k=2 (green), k=3 (blue), and k=4 (grey), and should be read on the left scale.

Each bipartition corresponds to a line from below (darker) to above (lighter), in agreement with (19). They are shown piled on each other in an additive way, with each one scaled by the factor $\frac{1}{4N^{\left(k\right)}}$, as for $E_{\tau }^{\mathrm{WAE}}\left[\rho \right]$. Thus, the gap between lines provides each individual measure integrated into one global measure. The top grey line provides the global measure $E_{E^{N}}^{\mathrm{WAE}}\left[\rho \right]$. Despite the different scales, note that for larger values of *T* this global measure reaches its maximum at the beginning of the dynamics. Despite these measures being monotones and having been re-scaled, we can note the different information provided by each one; for example, $E^{\mathrm{MW}}\left[\rho \right]$ is in a sense an average measure about the even filling of populations ($\rho_{ii}=\frac{1}{N}=\frac{1}{8},i=1,...,8$): $E^{\mathrm{MW}}\left[\rho \right]=\frac{4}{8}\left(1-\sum_{i=1}^{8}\rho_{ii}^{2}\right)\leq \frac{7}{16}=0.4375$. The maximum value is almost reached by *P. aestuarii* around 1ps, under T=77K, and *C. tepidum* almost reaches a value between 2−3ps. In another issue, $E_{E^{N}}^{\mathrm{WAE}}\left[\rho \right]$ exhibits larger values for certain bipartitions if the gap between lines are noticeable (see the upper one for such gap). Observing Figure 6 and Figure 7 panels (a) and (d), such gaps are present for BChl 8 in the beginning. Afterwards, they are apparently more spaced as a consequence of the averaging re-scaling. Note the dramatic differences imposed by the increase of *T*. In general, there is no apparently privileged order *k* for the bipartitions in terms of a larger entanglement provided by this measure.

In general, despite both being based in bipartitions, it could be said that $E_{E^{N}}^{\mathrm{WAE}}\left[\rho \right]$ comprises more entanglement cases than $E^{\mathrm{MW}}\left[\rho \right]$ due to being mainly oriented to the entanglement of single BChls with the remaining system. Regarding the maximum observed in the last measure in comparison with the minimum observed in $E_{E^{N}}^{\mathrm{WAE}}\left[\rho \right]$, this shows that each BChl remains entangled with the remainder at the beginning of the dynamics. Instead, a tiny entanglement between all possible bipartition appears followed by a remarkable increase, which then fades with the increase in temperature.

This brief review provides an illustrative vision of entanglement measures, as well as those composed of single measures such as $C_{\left\{kl\right\}}$ and $C_{\left\{k\right\}}$. Note that $E^{\mathrm{MW}}\left[\rho \right]$ and $E_{E^{N}}^{\mathrm{WAE}}\left[\rho \right]$ were selected as two bipartite emblematic measures regarding the entanglement between each BChl and its environment as well as between each pair of subgroups of BChls.

#### 2.3.2. The Maximum Fidelity with Respect to the Closest Separable State: An Authentic Multipartite Entanglement Measure

Until now the entanglement of the BChls has been characterized in terms of measures based on the bipartitions of the system. However, in this section we discuss the entanglement quantification of subsystems composed of three components in terms of fidelity with respect to the closest separable state. This is a subtle distinction inside the multipartite entanglement complexity, because while bipartition-based entanglement measures refer to entanglement regarding the overall set of BChls, subgroups of them can instead possibly involve quantum correlations independently of the remaining BChls. For the measure, this quantity should be able to detect fully separable states between the BChls in a subgroup of all of them.

We begin with several definitions. A density matrix σ is called fully separable if it can be written as the following convex combination [72]:

(20) $$\sigma =\sum q_{k}\sigma_{k}^{\left(1\right)}⊗\cdots ⊗\sigma_{k}^{\left(N\right)},\;\sum_{k}q_{k}=1,\;q_{i}\geq 0,$$

where the density operator $\sigma_{k}^{\left(i\right)}$ acts on the space of the *i*-th BChl. For an arbitrary multipartite mixed state ρ, the *maximum fidelity with respect to the closest separable state* is defined as

(21) $$\Lambda \left(\rho \right)=\max_{\sigma \in S}{\mathrm{Tr}}^{2}\left(\sqrt{\sqrt{\rho }\sigma \sqrt{\rho }}\right),$$

where *S* stands for the set of fully-separable density matrices [73]. The quantity (21) is bounded between 0 and 1, and the largest value is attained for states of the form (20). Note that the closer Λ is to zero, the more entangled the state is. For this reason, sometimes it is useful to use instead the *geometric measure of entanglement* defined as $E_{G}\left(\rho \right)=1-\Lambda \left(\rho \right)$ [74]. Thus, this measure is free of the partitioned principle to understand entanglement. Instead, it recurs to the separability concept and a metric approach measuring the minimum geometrical distance of each state to the closest separable state through the fidelity measure. This natural measure is in the scale [0,1], from the closest state to the farthest one to the separable state subspace in the Hilbert space regarded.

In the case of two-qubit mixed systems, Λ can be evaluated in terms of concurrence [74]. However, a closed expression in the multipartite scenario is not known even for pure states, and one has to rely on numerical procedures. Our purpose is to analyse the maximum fidelity with respect to the closest separable state Λ for subsystems compounded of three BChls, whose density matrix is in general mixed. Accordingly, we implemented a numerical algorithm which first computed a generalized Schmidt decomposition for multi-qubit pure states, then optimized over all the possible decompositions of a mixed state using Uhlmann’s theorem [73].

**Figure 8:**
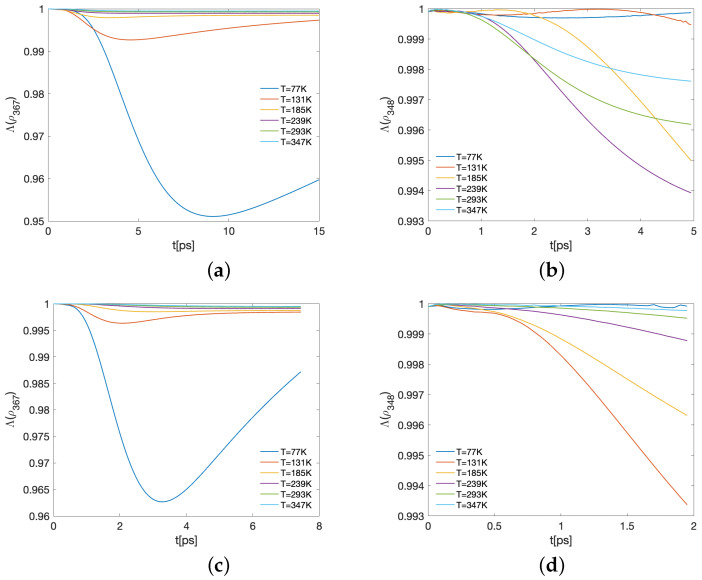
Time-evolution of the maximum fidelity with respect to the closest separable state of the three BChls 3,6,7 (first column) and 3,4,8 (second column) in *P. aestuarii* for (**a**,**b**) $\lambda_{k}=35\;{\mathrm{cm}}^{-1}$ and (**c**,**d**)$\;\lambda_{k}=65\;{\mathrm{cm}}^{-1}$.

**Figure 9:**
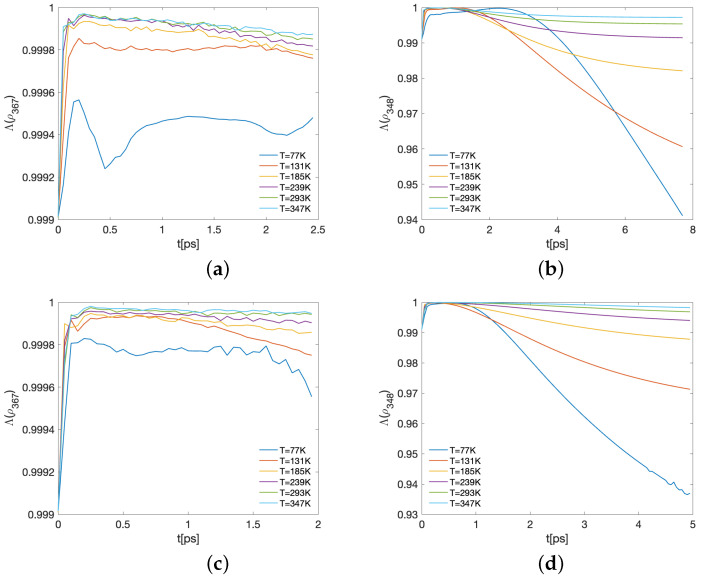
Time-evolution of the maximum fidelity with respect to the closest separable state of the three BChls 3,6,7 (first column) and 3,4,8 (second column) in *C. tepidum* for (**a**,**b**) $\lambda_{k}=35\;{\mathrm{cm}}^{-1}$ and (**c**,**d**)$\;\lambda_{k}=65\;{\mathrm{cm}}^{-1}$.

Representative results of the Λ time-evolution are shown in Figure 8 and Figure 9 for several values of the temperature. In each case, panels (a,c) correspond to the BChls 3, 6, and 7, commonly the three with the largest ending populations, for $\lambda_{k}=35\;{\mathrm{cm}}^{-1},65\;{\mathrm{cm}}^{-1}$, respectively, while panels (b,d) show the subsystem compounded with the energy-exit BChls, 3 and 4, together with the main entry energy BChl, 8. This correlates the main entry and the supposed main exits in the spectroscopy observations. In this case, $\lambda_{k}=35\;{\mathrm{cm}}^{-1},65\;{\mathrm{cm}}^{-1}$ with respect to the panels. In both cases, the three-qubit reduced density matrix is computed as $\rho_{\left\{j,k,\ell \right\}}={\mathrm{Tr}}_{\left\{j,k,\ell \right\}}\left(\rho \right)$. As before, the partial trace is taken over the complement of the set {j,k,ℓ}. In all the cases, $\gamma_{k}=50\;{\mathrm{cm}}^{-1}$ and the time-interval shown corresponds with when the more relevant entanglement behaviour happens. We first note that the initial state $\rho_{\mathrm{FRET}}^{8}$ (5) is not fully-separable, as it is the convex combination of the eigenvectors $|\epsilon_{k}\rangle$, which in general are entangled. Thus, in general, any partition of the initial state attains a non-vanishing entanglement degree. As a reference, the Λ-values for the eigenvectors are shown in Table 1.

For later times, the state is in general close to a fully-separable state. This is particularly evident in Figure 9a,c, where the values in the vertical axis fluctuate around the worked numerical precision. Nevertheless, in the remaining cases the model allows us to produce a more entangled state depending on the temperature. For instance, in Figure 8a,c, the higher the temperature, the more entangled the BChl state of 3, 6, and 7 becomes, reaching the lowest Λ for T=77K at around 9.05ps and 3.2ps for $\lambda_{k}=35\;{\mathrm{cm}}^{-1}$ and $\lambda_{k}=65\;{\mathrm{cm}}^{-1}$, respectively. In the case of BChls 3, 4, and 8, panels b,d of Figure 8 and Figure 9 show dependence of the entanglement on both the temperature and $\lambda_{k}$.

#### 2.3.3. Entanglement Measures Using Bipartitions against Authentic Multipartite Measures in FMO

It is well known that in the multipartite case there is no universal measure to quantify entanglement. In this sense, each one of the proposed measures provides different information on the non-local correlations. Accordingly, no unique maximally entangled state can be identified in general [75]. The present analysis has been focused on three-qubit states, which stand for the simplest multipartite entanglement scenario and represent, to our knowledge, one of the first steps in the characterization of entanglement in the FMO systems beyond measures based on bipartitions.

The current measure, the maximum fidelity with respect to the closest separable state (or alternatively the geometric measure of entanglement), has not been used before in the analysis of FMO entanglement, and genuinely shows the clear existence of entanglement among three BCHls (used here as an example because of the algorithmic complexity of higher subsystems). This measure is not directly shown by other measures previously used in the literature, which in fact provide other entanglement features, typically only involving bipartitions such as those constructed in Section 2.3.1.

Despite being tiny (near to one), again, a cumulative effect could be expected for the bipartition-based versions. For instance, $E_{A|B}^{N}\left[\rho \right]$ reflects the entanglement of several *k*-sized bipartions in an additive way (Figure 6 and Figure 7). For *P. aestuarii*, they clearly grow at the end before decreasing, particularly for the lowest temperatures. Those for k=1 provide higher contributions, mainly due to the lower number of cases. For *C. tepidum*, this behaviour grows again for a second time at the very end. Nevertheless, for both species the main contribution grows as *k* grows. Instead, the global measure $E^{\mathrm{MW}}\left[\rho \right]$ exhibits a single peak behaviour in its growth, commonly delayed with respect to $E_{A|B}^{N}\left[\rho \right]$. This measure, unlike the previous ones, only considers the entanglement of each BChl, with the rest in agreement with (14). In both measures and both species, a remainder entanglement appears due to the tiny correlations between bipartitions or the entire sets of BChls. When the maximum fidelity with respect to the closest separable state is observed between BChls of a subset of the BChls in the monomer, the tiny entanglement there is verified (although we show only the authentic entanglement among the three emblematic groups of BChls).

Thus, there is a global climax when all single entanglements of each BChl with the remaining ones is reached (provided by $E^{\mathrm{MW}}\left[\rho \right]$, the red dashed line in Figure 6 and Figure 7). Such a climax does not equate to that reached when most bipartitions become entangled on average (indicated by $E_{E^{N}}^{\mathrm{WAE}}\left[\rho \right]$ in Figure 6 and Figure 7). The last condition appears prevalent at the end of the dynamics in different strengths as a function of *T*. In another trend, authentic tripartite entanglement for BChls 3, 6, and 7 and 3, 4, and 8 initially increases, then decreases again during the dynamics (Λ[ρ] decreases) as a function of *T* and at different timescales, as Figure 8 and Figure 9 suggest. All of these are observed behaviours resulting from the different entanglement measures used.

In any case, it is noticeable that a fundamental feature of GBS, as entanglement is, can be finally tracked with the convergence of experimental techniques, theoretical physics, mathematical modelling, and computational biology [76]. This remarkable effort to understand the more basic quantum behaviour behind the photosynthetic processes for such bacteria exhibits the importance of multidisciplinary research merging biology, physics, mathematics, and computer science [30].

### 2.4. State Transference Analysis

This section analyses the state’s transference through the dynamic process. In fact, the initial state $\rho_{\mathrm{FRET}}^{8}$ is distributed through the localization states with the three central populations: $\rho_{8,8}\left(0\right)=0.707,\rho_{1,1}\left(0\right)=0.216,\rho_{2,2}\left(0\right)=0.065$ for *P. aestuarii* and $\rho_{8,8}\left(0\right)=0.703,\rho_{5,5}\left(0\right)=0.080,\rho_{1,1}\left(0\right)=0.065$ for *C. tepidum*. The analysis tracks such states distributed on the set of BChls in a different arrangement. Thus, by considering all BChls permutations, we have

(22) $$\begin{array}{c} \Pi =\left\{\pi_{i}|i=1,...,8!\right\}=\left(\left\{1,2,...,7,8\right\},\left\{1,2,...,8,7\right\},...,\left\{2,1,3,...8\right\},...,\left\{8,7,...,1\right\}\right) \end{array}$$

ordered place by place, which means that each subgroup of permutations is ordered first by its first position inside, again by the second, and so on. There are 8!=40,320 different permutations. Then, we obtain the fidelity $F_{\pi_{i}}$ for each permutation:

(23) $$\begin{array}{c} F_{\pi_{i}}\left(t\right)={\mathrm{Tr}}^{2}\left(\sqrt{\sqrt{\rho_{\mathrm{FRET}}^{8}}\;\rho \left(t\right)\;\sqrt{\rho_{\mathrm{FRET}}^{8}}}\right) \end{array}$$

and as function of *t* through the evolution. In each *t*, we obtain the maximum fidelity on the entire set of BChls permutations: $F_{\Pi }\left(t\right)=\max_{\Pi }F_{\pi_{i}}\left(t\right)$. Clearly, $F_{\Pi }\left(t\right)$ occurs in each *t* for a different permutation with the time interval. As is well-known, fidelity is a positive valued measure providing a comparison between two states, pure or mixed, obtained as the inner product between them. When both states fit perfectly, this measure becomes one; otherwise, if states are orthogonal (which is understood as the most different possible state), it drops to zero. By tracking the time that represents the best fitting of the initial state as the energy excitation moves into the whole system, we can try to identify which permutation of BChls best fits that initial state at each time. In a sense, this is a discrete comparison of the continuous forms of a wavefunction when tunnelling through a potential obstacle. The outcomes of such tracking are analysed in the next subsections.

#### 2.4.1. Comparative: Input versus Output States

**Figure 10:**
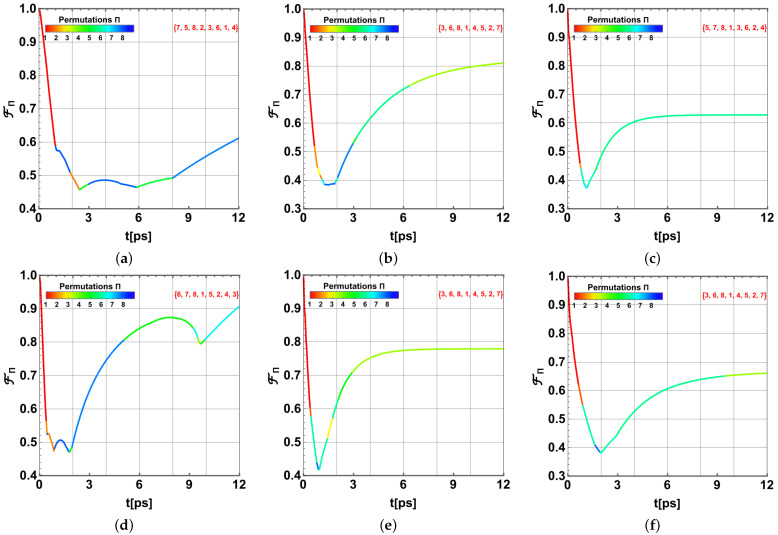
State transference comparison in terms of $F_{\Pi }$: (**a**–**c**) for $\lambda_{k}=35\;{\mathrm{cm}}^{-1}$ and (**d**–**f**) for $\lambda_{k}=65\;{\mathrm{cm}}^{-1}$. Each set of three shows T=77,181,293K, respectively. In all cases, $\gamma_{k}=50\;{\mathrm{cm}}^{-1}$ for *P. aestuarii*. The colour in each segment shows the most similar permutation to the initial state $\rho_{\mathrm{FRET}}^{8}$ in agreement with the colour bar indicating the first number in the permutation.

**Figure 11:**
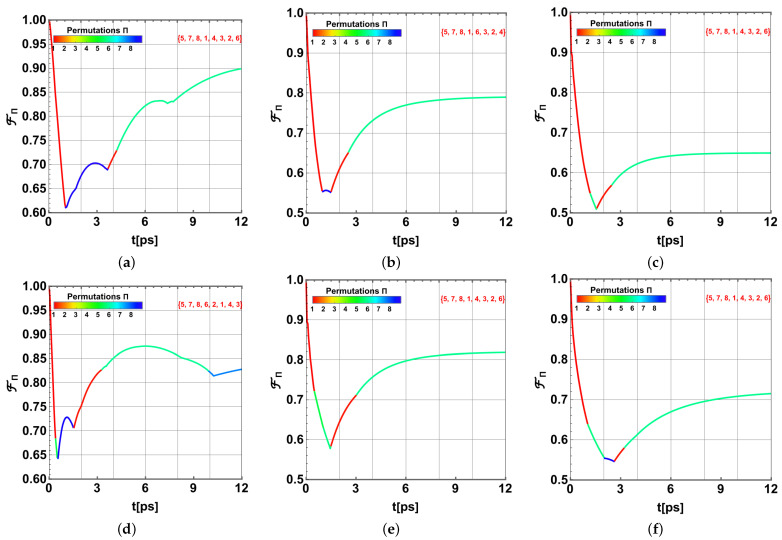
State transference comparison in terms of $F_{\Pi }$: (**a**–**c**) for $\lambda_{k}=35\;{\mathrm{cm}}^{-1}$ and (**d**–**f**) for $\lambda_{k}=65\;{\mathrm{cm}}^{-1}$. Each set of three shows T=77,181,293K, respectively. In all cases, $\gamma_{k}=50\;{\mathrm{cm}}^{-1}$ and for *C. tepidum*. The colour in each segment shows the most similar permutation to the initial state $\rho_{\mathrm{FRET}}^{8}$ in agreement with the colour bar indicating the first number in the permutation.

The analysis outcomes are presented by remarking on the permutation $\pi_{i}$ nearest to the initial state as measured by $F_{\Pi }\left(t\right)$. Thus, Figure 10 (*P. aestuarii*) and Figure 11 (*C. tepidum*) show the state transference dynamics in t∈[0,12]ps for the representative temperatures T=77,185,293K. In each case, panels (a–c) correspond to $\lambda_{k}=35\;{\mathrm{cm}}^{-1}$ and panels (d–f) correspond to $\lambda_{k}=65\;{\mathrm{cm}}^{-1}$. In all cases, $\gamma_{k}=50\;{\mathrm{cm}}^{-1}$. In each plot, each different coloured segment corresponds to the closest permutation carrying out a similar state to the initial $\rho_{\mathrm{FRET}}^{8}$. Colours are reported in the colour bar at the top. The scale reports the initial BChls in the permutation in agreement with the order settled in (22), and each unitary interval in the bar comprises 7! permutations.

In each panel, the dominant asymptotic permutation is reported in red on the top right. Thus, considering that the initial permutation is {1,2,...,8}, we observe that for *P. aestuarii* the initial dominant excitations in BChls 8, 1, and 2 finally move on (4,7,5), (7,3,6), (3,6,7), or (4,5,7), respectively, depending on the set of parameters $T,\lambda_{k}$ (those BChls in the original positions of the initial excitations). For *C. tepidum*, the initial dominant excitations in BChls 8, 5, and 1 finally move on (6,4,5), (4,6,5), or (3,2,5), respectively.

Interesting facts arise here. First, note the similarities between the corresponding panels in Figure 10 and Figure 11. In addition, the speedup in the process is imposed by temperature, together with a slower recovery into an similar state to the initial one ($F_{\Pi }$ lowering). A similar effect is produced by the increase of the $\lambda_{k}$ value. Note that for the lower temperature the similarity transitions are more acute. All cases in the dynamics exhibit a kind of excitation dispersion followed by a regrouping on other BChls, which is more uniform for *C. tepidum* than for *P. aestuarii*.

#### 2.4.2. Analysis of Main Excitation Paths

**Figure 12:**
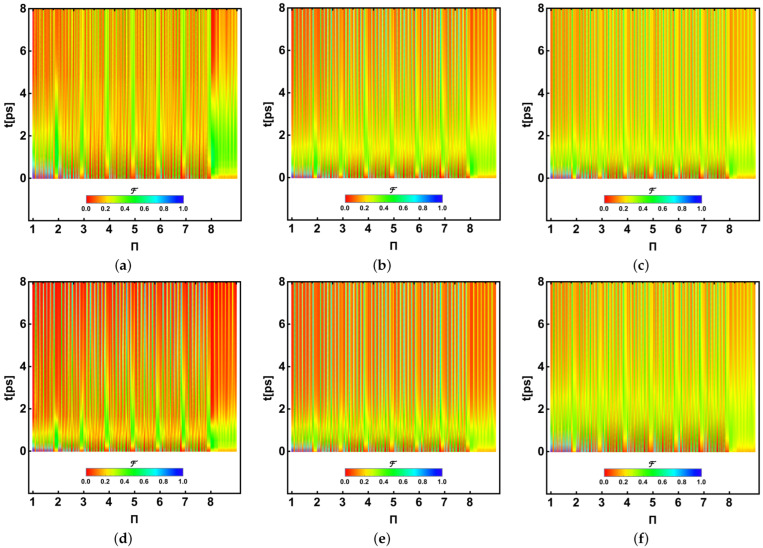
Similarity state transference evolution comparison for *P. aestuarii* in t∈[0,8] ps for each permutation in Π coloured in agreement with their fidelity F in agreement with the colour scale in the bottom: (**a**–**c**) for $\lambda_{k}=35\;{\mathrm{cm}}^{-1}$ and (**d**–**f**) for $\lambda_{k}=65\;{\mathrm{cm}}^{-1}$. Each set of three shows T=77,181,293K, respectively. In all cases, $\gamma_{k}=50\;{\mathrm{cm}}^{-1}$. The horizontal scale for permutations contains the 8! cases ordered and referred to by the first digit.

**Figure 13:**
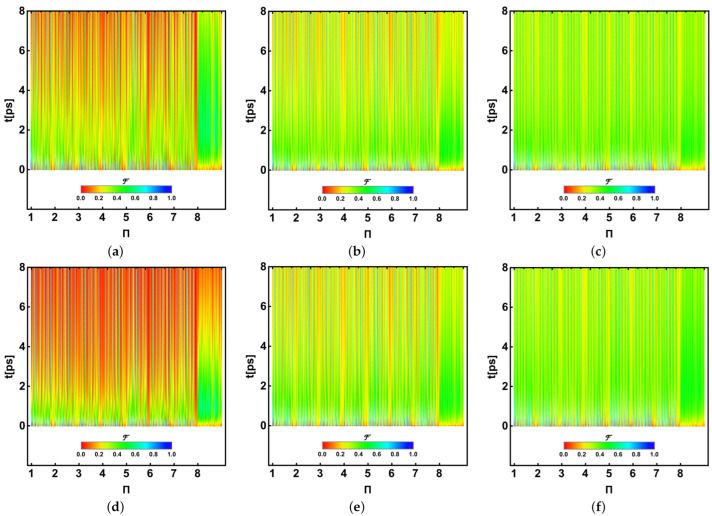
Similarity state transference evolution comparison for *C. tepidum* in t∈[0,8] ps for each permutation in Π coloured in agreement with their fidelity F in agreement with the colour scale in the bottom: (**a**–**c**) for $\lambda_{k}=35\;{\mathrm{cm}}^{-1}$ and (**d**–**f**) for $\lambda_{k}=65\;{\mathrm{cm}}^{-1}$. Each set of three shows T=77,181,293K, respectively. In all cases, $\gamma_{k}=50\;{\mathrm{cm}}^{-1}$. The horizontal scale for permutations contains the 8! cases ordered and referred to by the first digit.

In an alternative view, we provide a global vision of the analysis of the whole fidelities for all permutations. Figure 12 (*P. aestuarii*) and Figure 13 (*C. tepidum*) show the fidelities calculated for the entire 8! permutations in the horizontal axis, evolving on the vertical one for the first 8ps. Each fidelity is shown as a point in agreement with the colour bar scale at the bottom, with 0 (red) and 1 (blue) representing the fidelity. In each figure, panels (a–c) correspond to $\lambda_{k}=35\;{\mathrm{cm}}^{-1}$ and T=77,181,293K and (d-f) to $\lambda_{k}=65\;{\mathrm{cm}}^{-1}$ for the same corresponding *T*. In all cases, $\gamma_{k}=50\;{\mathrm{cm}}^{-1}$. Points are horizontally arranged as a function of the permutation using a representative scale showing the integers 1–8, the first number in the permutation; thus, each unitary space contains 7! permutations, meaning that the bluest vertical traces show the most likely permutations to which the initial excitation shape is moving.

Plots should be interpreted as follows. Note the permutation 1 (1,…,8 in fact) at t=0 ps on the left in blue, indicating the initial state. This initial excitation evolves by dispersion to reach a similar structure on other BChls or permutations. Red zones indicate fewer likelihood distributions where that initial excitation shape could be located, while the bluest or greenest ones show higher likelihood. Again, a speedup can be observed for the largest $\lambda_{k}$ with the lowest *T*. The similarity between the plots for larger temperatures is again observed. Upper green or blue bands denote the main state transference to certain BChls (3, 4, 7), as indicated in the previous subsection, despite one permutation being optimal, as indicated in Figure 10 and Figure 11. Despite not being identical, this tracing analysis of the initial state distribution provides insight into the more feasible paths being followed by excitation through the set of eight BChls when energy arrives crossing the structure. Note the fast decay of the eighth BChl in the lead of this transition. In addition, it should be noted that for *P. aestuarii* (reddest) the final state transference is higher, which is preserved through fewer BChl combinations (green or blue) than for *C. tepidum* (greenest/yellowest), where the exit similarity is partial (in agreement with Figure 10 and Figure 11). Nevertheless, this better-defined final transference is dependent on the temperature (lower) and more noticeable for $\lambda_{k}=65\;{\mathrm{cm}}^{-1}$. This analysis suggests that the tunnelling is clearly limited by the temperature increase. Nonetheless, this analysis shows that for lower temperatures the tunnelling phenomenon appears strongly if $\lambda_{k}$ increases, otherwise a dispersion effect appears.

## 3. Materials and Methods

### 3.1. Data Collection

Experimental data introduced in the analysis are achieved through the Hamiltonians reported in Appendix A for both species, *P. aestuarii* and *C. tepidum*. Those Hamiltonians were obtained by combining theory and experiment in spectroscopy including the eighth BChls. Other key structural parameters involved as $r_{\mathrm{trap}}=1\;{\mathrm{ps}}^{-1},\lambda_{k}=35,65\;{\mathrm{cm}}^{-1}$ and $\gamma_{k}=50\;{\mathrm{cm}}^{-1}$ are considered departing from the best spectroscopy observations available. Such experimental information is introduced in the HEOM modelling to reach a time-dependent depiction of the density matrix ρ(t) for the BChls system. Using spectroscopy units implied that energy is reported in ${\mathrm{cm}}^{-1}$ using the conversion factor 200πℏc. In addition, the conversion between ${\mathrm{cm}}^{-1}$ and $s^{-1}$ is reached through the factor 200πc.

### 3.2. Time-Dependent Density Matrix

HEOM method was implemented under the supervector-superoperator procedure depicted in Section 2.1.4. Implementation of HEOM method was programmed using *Mathematica*. The basic simulations to reach ρ(t) with HEOM, a depth D=4 was used with processing power in the range of 3.4 GHz and implementing parallel computing with twelve processing cores. Each computer process with a fixed set of parameters took around 10 h considering time simulations of 15ps to reach the equilibrium of populations in $\rho_{S}$.

Using the last HEOM implementation with D=4, together with the parameters $r_{\mathrm{trap}}=1\;{\mathrm{ps}}^{-1},\gamma_{k}=50\;{\mathrm{cm}}^{-1}$, and the pair of values $\lambda_{k}=35,65\;{\mathrm{cm}}^{-1}$ (k=1,...,8), a set of computer simulations between t∈[0,15]ps were performed for both species, *P. aestuarii* and *C. tepidum*. Such a set was obtained by considering a wide group of representative temperatures: T=77,131,185,239,293,347K. In addition, the overall analysis considered the use of $\rho \left(0\right)=\rho_{\mathrm{FRET}}^{8}$ as the initial condition. Each simulation was stored as an individual file of complex-valued data regarding each entry of ρ(t) for the times considered in t∈[0,15]ps. The numerical time-step for calculations being used was $\delta t=10^{-4}\;\mathrm{ps}$, despite, only data with a time-step of $\Delta t=10^{-3}\;\mathrm{ps}$ were stored. Those data synthetically and partially represented in Figure 4 and Figure 5 (only the populations $\rho_{ii}\left(t\right)$ are plotted there) became the input for further analyses.

### 3.3. Entanglement Evolution Analysis

Regarding the ρ(t) values stored, several entanglement measures were obtained for each corresponding time. For $E_{A|B}^{N}\left[\rho \right]$ (and then for $E_{E^{N}}^{\mathrm{WAE}}\left[\rho \right]$) and $E^{\mathrm{MW}}\left[\rho \right]$ own *Mathematica* programming procedures were constructed regarding the overall bipartitions needed. Those procedures let to reach the last measures for the corresponding discrete times. Similarly, to reach each $\Lambda (\rho_{jk\ell }\left(t\right))$ considered in the analysis, the corresponding Uhlmann’s procedure [73] was programmed using *Matlab*.

### 3.4. State Transference Evolution Analysis

State transference analysis has required to transform each ρ(t) stored by exchanging the order of BChls in agreement with each element of the set Π. The procedure was directly constructed in *Mathematica* to then get $F_{\pi_{i}}\left(t\right)$ for each element in the set, and in each discrete time for our original data in ρ(t). Finally, the maximum value, $F_{\Pi }\left(t\right)$ in each time is easily obtained to become represented in Figure 10 and Figure 11. Instead, Figure 12 and Figure 13 are complete representations of each $F_{\pi_{i}}\left(t\right)$ through the time.

## 4. Conclusions

Quantum features are undoubtedly present in living systems. We are still learning how these features set important aspects of life survival. With even higher complexity than chemical systems, the task is very intricate because it needs to pass through several size orders. In such circumstances, computational biology is advisable as an important element gathering efforts from several disciplines, including physics, chemistry, mathematics, and computer science, leading to specialized computer simulations that can describe the meaningfulness of these features for life.

For FMO, energy transition through LHC exhibits clear quantum features such as entanglement among BChls, together with state tunnelling crossing the set of BChls to reach the RC. The analysis of the behaviour of these quantum features requires experimental work developed in the spectroscopy domain as well as computer modelling to perform simulations of the complex processes involved in their dynamics. Such combined approaches are settled in the terrain of computational biology to obtain insight into relevant features. The current work extends several traditional approaches to analyse the quantum features of entanglement and state transference.

By introducing alternative or more elucidated entanglement measures for the set of eight subsystems, a clearer understanding of the phenomenon is pursued. Note that due to the current limitations of entanglement measures for larger mixed states, an approach based on bipartitions becomes useful; however, each type of measure provides different information. In addition, authentic multipartite entanglement beyond bipartite becomes present, as our analysis based on the fidelity with respect to the closest separable state has shown.

In this sense, multipartite entanglement must be understood from different viewpoints beyond the bipartite approach. The complexity of this quantum phenomenon is reflected in the fact that several entanglement measures have been proposed, each one considering particular aspects of non-locality. In the present work, we have considered the maximum overlap with respect to the closest separable state as a natural entanglement measure, as it provides information concerning the likeness of a given mixed state with one that is fully separable. We focused on two particular cases: the three BChls with the largest populations, and BChls 3, 4, and 8, which are related to the main BChl exit and entry. Whereas most of the measures used in the literature are computed as the averages of bipartite measures, this function is able to quantify the entanglement of subsystems compounded with three or more qubits. In this context, we considered partitions of three chromophores and computed Λ[ρ] using a numerical algorithm [73].

Nevertheless, the same study could be accomplished for any number of BChls traced out the general state. Our numerical simulations showed that the state of the system is close to being in a fully-separable state at most times, even though there are cases where a non-vanishing degree of entanglement is achieved. Our study represents a step forward in the analysis of the multipartite entanglement of three or more BChl subsystems.

For state transference, models based on the tracking of the initial state distribution through BChls have allowed us to identify a process with fidelities above 60% for the worst cases, sometimes reaching nearly 80%. In particular, the approach taken in this work to identifying the initial state in all possible permutations of the site basis elements has allowed us to identify its transition through the evolved state in this complex feature, again strongly supported by a computational approach regarding a post-experimental simulation analysis on the biological FMO system.

Superposition, entanglement, and tunnelling are key aspects of the quantum world. Recently, it has been noticed that entangled systems enlarge the coherence time needed for quantum process [71,77]. Thus, by extending the entanglement, it appears that coherence becomes preserved over larger times, which provides the possibility of computing the energy transference more accurately. In such research, each time larger system sizes are used, implementing an atom in a superposition of hundreds of locations, the quantum coherence becomes extended on the scale of seconds. If a similar mechanism happens in living systems the related quantum effects could play a key role in daily survival.

## Figures and Tables

**Table 1:** The maximum fidelity with respect to the closest separable state of the eigenvectors $|\epsilon_{k}\rangle$ for the BChls *P. aestuarii* (centre) and *C. tepidum* (right).

$|\epsilon_{k}\rangle$	$\Lambda_{P.\;\mathrm{aestuarii}}(|\epsilon_{k}\rangle \langle \epsilon_{k}|)$	$\Lambda_{C.\;\mathrm{tepidum}}(|\epsilon_{k}\rangle \langle \epsilon_{k}|)$
$|\epsilon_{1}\rangle$	0.9888	0.9909
$|\epsilon_{2}\rangle$	0.7050	0.7683
$|\epsilon_{3}\rangle$	0.9287	0.9327
$|\epsilon_{4}\rangle$	0.7159	0.8604
$|\epsilon_{5}\rangle$	0.5719	0.9397
$|\epsilon_{6}\rangle$	0.8315	0.8275
$|\epsilon_{7}\rangle$	0.8110	0.9454
$|\epsilon_{8}\rangle$	0.8292	0.9235

## Data Availability

Not applicable.

## Abbreviations

The following abbreviations are used in this manuscript:

BChl	Bacteriochlorophyl
EET	Excitation Energy Transfer
FMO	Fenna–Matthews–Olson complex
FRET	Förster resonance energy transfer
GSB	Green Sulphur Bacteria
HEOM	Hierarchical Equations of Motion
LHC	Light Harvesting Complexes
RC	Reaction Centre

## Appendix A. Dipole–Dipole Hamiltonians for *N* = 7 and *N* = 8 BChls

Dipole–dipole interactions among the eight BChls within each monomer in the FMO rise the Hamiltonian $H_{S}$. As obtained by spectroscopy studies and theoretical modelling analysis, $H_{S}$ for the eight BChls model for *C. tepidum* [24] is

(A1) $$\begin{array}{c} H_{S}=\left(\begin{array}{cccccccc} 255 & -87 & 4.2 & -5.2 & 5.5 & -14 & -6.1 & 21\\ -87 & 355 & 28 & 6.9 & 1.5 & 8.7 & 4.5 & 4.2\\ 4.2 & 28 & 0 & -54 & -0.2 & -7.6 & 1.2 & 0.6\\ -5.2 & 6.9 & -54 & 150 & -62 & -16 & -51 & -1.3\\ 5.5 & 1.5 & -0.2 & -62 & 320 & 60 & 1.7 & 3.3\\ -14 & 8.7 & -7.6 & -16 & 60 & 425 & 29 & -7.9\\ -6.1 & 4.5 & 1.2 & -51 & 1.7 & 29 & 225 & -9.3\\ 21 & 4.2 & 0.6 & -1.3 & 3.3 & -7.9 & -9.3 & 280 \end{array}\right) \end{array}$$

with a constant diagonal offset of 12,150 ${\mathrm{cm}}^{-1}$. In the same model with eight BChls for *P. aestuarii* [5], $H_{S}$ is

(A2) $$\begin{array}{c} H_{S}=\left(\begin{array}{cccccccc} 424 & 94.8 & 5.5 & -5.9 & 7.1 & -15.1 & -12.2 & 39.5\\ 94.8 & 320 & 29.8 & 7.6 & 1.6 & 13.1 & 5.7 & 7.9\\ 5.5 & 29.8 & 0 & -58.9 & -1.2 & -9.3 & 3.4 & 1.4\\ -5.9 & 7.6 & -58.9 & 334 & -64.1 & -17.4 & -62.3 & -1.6\\ 7.1 & 1.6 & -1.2 & -64.1 & 424 & 89.5 & -4.6 & 4.4\\ -15.1 & 13.1 & -9.3 & -17.4 & 89.5 & 305 & 35.1 & -9.1\\ -12.2 & 5.7 & 3.4 & -62.3 & -4.6 & 35.1 & 298 & -11.1\\ 39.5 & 7.9 & 1.4 & -1.6 & 4.4 & -9.1 & -11.1 & 548 \end{array}\right) \end{array}$$

with a constant diagonal offset of 12,178 ${\mathrm{cm}}^{-1}$. These Hamiltonians were obtained using the charge density coupling method [9,25] considering a standard protonation pattern [30].

## References

[1] Grisolia G., Fino D., Lucia U. (2020). Thermodynamic optimisation of the biofuel production based on mutualism. Energy Rep..

[2] Lucia U., Grisolia G. (2021). Biofuels Analysis Based on the THDI Indicator of Sustainability. Front. Energy Res..

[3] Lucia U., Grisolia G. (2021). Biofuels from Micro-Organisms: Thermodynamic Considerations on the Role of Electrochemical Potential on Micro-Organisms Growth. Appl. Sci..

[4] Hauska G., Schoedl T., Remigy H., Tsiotis G. (2001). The reaction center of green sulfur bacteria. Biochim. Biophys. Acta.

[5] Schmidt am Busch M., Müh F., Madjet M.E.A., Renger T. (2011). The Eighth Bacteriochlorophyll Completes the Excitation Energy Funnel in the FMO Protein. J. Phys. Chem. Lett..

[6] Croce R., van Grondelle R., van Amorengen H., van Stokkum I. (2018). Light Harvesting in Photosynthesis.

[7] Leibl W., Trissl H.W. (1990). Relationship between the fraction of closed photosynthetic reaction centers and the amplitude of the photovoltage from light-gradient experiments. Biochim. Biophys. Acta.

[8] Blankenship R.E. (2014). Molecular Mechanisms of Photosynthesis.

[9] Adolphs J., Renger T. (2006). How proteins trigger excitation energy transfer in the FMO complex of green sulfur bacteria. Biophys. J..

[10] Müh F., Madjet M.E.A., Adolphs J., Abdurahman A., Rabenstein B., Ishikita H., Knapp E.W., Renger T. (2007). *α*-Helices direct excitation energy flow in the Fenna-Matthews-Olson protein. Proc. Natl. Acad. Sci. USA.

[11] Camacho A. (2009). Sulfur Bacteria. Encyclopedia of Inland Waters.

[12] Beatty J.T., Overmann J., Lince M.T., Manske A.K., Lang A.S., Blankenship R.E., Dover C.L.V., Martinson T.A., Plumley F.G. (2005). An obligately photosynthetic bacterial anaerobe from a deep-sea hydrothermal vent. Proc. Natl. Acad. Sci. USA.

[13] Blankenship R.E., Olson J.M., Miller M. (1995). Anoxygenic Photosynthetic Bacteria—Antenna Complexes from Green Photosynthetic Bacteria.

[14] Alexander B., Andersen J.H., Cox R.P., Imhoff J.F. (2002). Phylogeny of green sulfur bacteria on the basis of gene sequences of 16S rRNA and of the Fenna-Matthews-Olson protein. Arch. Microbiol..

[15] Saer R.G., Satnytskyi V., Magdaong N.C., Goodson C., Savikhin S., Blankenship R.E. (2017). Probing the excitonic landscape of the *Clorobaculum tepidum* Fenna-Matthews-Olson (FMO) complex: A mutagenesis approach. Biochim. Biophys. Acta.

[16] Maiuri M., Ostroumov E.E., Saer R.G., Blankenship R.E., Scholes G.D. (2018). Coherent wavepackets in the Fenna-Matthews-Olson complex are robust to excitonic-structure perturbations caused by mutagenesis. Nat. Chem..

[17] Kramer T., Rodriguez M. (2017). Two-dimensional electronic spectra of the photosynthetic apparatus of green sulfur bacteria. Sci. Rep..

[18] Maity S., Kleinekathöfer U. (2022). Recent progress in atomistic modeling of light-harvesting complexes: A mini review. Photosynth. Res..

[19] Ravi S.K., Swainsbury D.J.K., Singh V.K., Ngeow Y.K., Jones M.R., Tan S.C. (2017). A Mechanoresponsive Phase-Changing Electrolyte Enables Fabrication of High-Output Solid-State Photobiolectrochemical Devices from Pigment-Protein Multilayers. Adv. Mater..

[20] Vattay G., Kauffman S. (2013). Evolutionary Design in Biological Quantum Computing. arXiv.

[21] Mohseni M., Shabani A., Lloyd S., Rabitz H. (2014). Energy-scales convergence for optimal and robust quantum transport in photosynthetic complexes. J. Chem. Phys..

[22] Fenna R.E., Matthews B.W. (1975). Chlorophyll arrangement in a bacteriochlorophyll protein from Chlorobium limicola. Nature.

[23] Fenna R.E., Matthews B.W., Olson J.M. (1978). Structure of a Bacteriochlorophyll a-Protein from the Green Photosynthetic Bacterium Prosthecochloris aestuarii. J. Mol. Biol..

[24] Kell A., Blankenship R.E., Jankowiak R.J. (2016). Effect of Spectral Density Shapes on the Excitonic Structure and Dynamics of the Fenna-Matthews-Olson Trimer From Chlorobaculum Tepidum. J. Phys. Chem..

[25] Adolphs J., Müh F., Madjet M.E.A., Renger T. (2008). Calculation of pigment transition energies in the FMO protein. Photosynth. Res..

[26] Sarovar M., Ishizaki A., Fleming G., Whaley K. (2010). Quantum entanglement in photosynthetic light-harvesting complexes. Nat. Phys..

[27] Bengtson C., Stenrup M., Sjöqvist E. (2018). Quantum nonlocality in the excitation energy transfer in the Fenna-Matthews-Olson complex. arXiv.

[28] Palmieri B., Abramavicius D., Mukamel S. (2009). Lindblad equations for strongly coupled populations and coherences in photosynthetic complexes. J. Chem. Phys..

[29] Jeske J., Ing D.J., Plenio M.B., Huelga S.F., Cole J.H. (2015). Bloch-Redfield equations for modeling light-harvesting complexes. J. Chem. Phys..

[30] González-Soria B., Delgado F., Anaya-Morales A. (2020). Parametric Mapping of Quantum Regime in Fenna–Matthews–Olson Light-Harvesting Complexes: A Synthetic Review of Models, Methods and Approaches. Appl. Sci..

[31] Tronrud D.E., Wen J., Gay L., Blankenship R.E. (2009). The structural basis for the difference in absorbance spectra for the FMO antenna protein from various green sulfur bacteria. Photosyn. Res..

[32] Wen J., Zhang H., Gross M.L., Blankenship R.E. (2011). Native Electrospray Mass Spectrometry Reveals the nature and Stoichiometry of Pigments in the FMO Photosynthesis Antenna Protein. Biochemistry.

[33] Hayes D., Engel G.S. (2011). Extracting the Excitonic Hamiltonian of the Fenna-Matthews-Olson Complex Using Three-Dimensional Third-Order Electronic Spectroscopy. Biophys. J..

[34] Camara-Artigas A., Blankenship R.E., James Allen P. (2003). The structure of the FMO protein from Chlorobium tepidum at 2.2Åresolution. Photosynth. Res..

[35] Engel G.S., Calhoun T.R., Read E.L., Ahn T.K., Mančal T., Cheng Y.C., Blankenship R.E., Fleming G.R. (2007). Evidence for wavelike energy transfer through quantum coherence in photosynthetic systems. Nature.

[36] Panitchayangkoon G., Hayes D., Fransted K.A., Caram J.R., Harel E., Wen J., Blankenship R.E., Engel G.S. (2010). Long-lived quantum coherence in photosynthetic complexes at physiological temperature. Proc. Natl. Acad. Sci. USA.

[37] Savikhin S., Buck D.R., Struve W.S. (1997). Oscillating anisotropies in a bacteriochlorophyll protein: Evidence for quantum beating between exciton levels. Chem. Phys..

[38] Wilkins D.M., Dattani N.S. (2015). Why Quantum Coherence Is Not Important in the Fenna–Matthews–Olsen Complex. J. Chem. Theory Comput..

[39] Fassioli F., Dinshaw R., Arpin P.C., Scholes G.D. (2014). Photosynthetic light harvesting: Excitons and coherence. J. R. Soc. Interface.

[40] van Amerongen H., Valkunas L., van Grondelle R. (2000). Photosynthetic Excitons.

[41] Förster T. (1959). Transfer Mechanisms of Electronic Excitation. Discuss. Faraday Soc..

[42] de Leon-Montiel R.J., Kassal I., Torres J.P. (2014). The Importance of Excitation and Trapping Conditions in Photosynthetic Environment-assisted Energy Transport. J. Phys. Chem. B.

[43] Rebentrost P., Mohseni M., Kassal I., Lloyd S., Aspuru-Guzik A. (2009). Environment-assisted quantum transport. N. J. Phys..

[44] Caruso F., Chin A.W., Datta A., Huelga S.F., Plenio M.B. (2009). Highly efficient energy excitation transfer in light-harvesting complexes: The fundamental role of noise-assisted transport. J. Chem. Phys..

[45] Mohseni M., Rebentrost P., Lloyd S., Aspuru-Guzik A. (2008). Environment-assisted quantum walks in photosynthetic energy transfer. J. Chem. Phys..

[46] Sahoo P.K., Benjamin C. (2020). Testing quantum speedups in exciton transport through a photosynthetic complex using quantum stochastic walks. arXiv.

[47] Brixner T., Stenger J., Vaswani H., Cho M., Blankenship R., Fleming G. (2005). Two-dimensional Spectroscopy of Electronic Couplings in Photosynthesis. Nature.

[48] Singh D., Dasgupta S. (2017). Role of Initial Coherence in Excitation Energy Transfer in Fenna-Matthews-Olson Complex. arXiv.

[49] Weidemüller M. (2009). There can be only one. Nat. Phys..

[50] Tanimura Y., Kubo R. (1989). Time Evolution of a Quantum System in Contact with a Nearly Gaussian-Markoffian Noise Bath. J. Phys. Soc. Japan.

[51] Kreisbeck C., Kramer T., Rodriguez M., Hein B. (2011). High-Performance Solution of Hierarchical Equations of Motion for Studying Energy Transfer in Light-Harvesting Complexes. J. Chem. Theory Comput..

[52] Pearle P. (2012). Simple derivation of the Lindblad equation. Eur. J. Phys..

[53] Plenio M.B., Huelga S.F. (2008). Dephasing assisted transport: Quantum networks and biomolecules. arXiv.

[54] Redfield A.G. (1965). The Theory of Relaxation Processes. Adv. Magn. Opt. Reson..

[55] Shabani A., Mohseni M., Rabitz H., Lloyd S. (2011). Optimal and Robust energy transfer in light-harvesting complexes Efficient simulation of excitonic dynamics in the non-perturbative and non-Markovian regimes. arXiv.

[56] Breuer H.P., Laine E.M., Piilo J., Vacchini B. (2016). Colloquium: Non-Markovian dynamics in open quantum systems. Rev. Mod. Phys..

[57] Jesenko S., Žnidarič M. (2012). Optimal number of pigments in photosynthetic complexes. N. J. Phys..

[58] Fleming G.R., Cho M. (1996). Chromophore-Solvent Dynamics. Annu. Rev. Phys. Chem..

[59] Cho M., Vaswani H.M., Brixner T., Stenger J., Fleming G.R. (2005). Exciton Analysis in 2D Electronic Spectroscopy. J. Phys. Chem. B.

[60] González-Soria B., Delgado F., Anaya-Morales A. (2021). Predicting entanglement and coherent times in FMO complex using the HEOM method. J. Phys. Conf. Ser..

[61] González-Soria B., Delgado F. (2023). Temperature dependence of entanglement and coherence in Fenna-Matthews-Olson complex. J. Phys. Conf. Ser..

[62] Peres A. (1996). Separability Criterion for Density Matrices. Phys. Rev. Lett..

[63] Horodecki M., Horodecki P., Horodecki R. (1996). Separability of mixed states: Necessary and sufficient conditions. Phys. Lett. A.

[64] González-Soria B., Delgado F. (2020). Quantum Entanglement in Fenna-Matthews-Olson Photosynthetic Light-Harvesting Complexes: A short Review of Analysis Methods. J. Phys. Conf. Ser..

[65] Baumgratz T., Cramer M., Plenio M.B. (2014). Quantifying Coherence. Phys. Rev. Lett..

[66] Horn R.A., Johnson C.R. (2013). Matrix Analysis.

[67] Caruso F., Chin A.W., Datta A., Huelga S.F., Plenio M.B. (2010). Entanglement and entangling power of the dynamics in light-harvesting complexes. Phys. Rev. A.

[68] Zhu J., Kais S., Aspuru-Guzik A., Rodriques S., Brock B., Love P.J. (2012). Multipartite quantum entanglement evolution in photosynthetic complexes. J. Chem. Phys..

[69] Meyer D.A., Wallach N.R. (2002). Global entanglement in multiparticle systems. J. Math. Phys..

[70] Thilagam A. (2012). Multipartite entanglement in the Fenna-Matthews-Olson (FMO) pigment-protein complex. J. Chem. Phys..

[71] Borras A., Majtey A.P., Plastino A.R., Casas M., Plastino A. (2009). Some features of the state-space trajectories followed by robust entangled four-qubit states during decoherence. Phys. Rev. A.

[72] Audretsch J. (2007). Entangled Systems: New Directions in Quantum Physics.

[73] Streltsov A., Kampermann H., Bruss D. (2011). Simple algorithm for computing the geometric measure of entanglement. Phys. Rev. A.

[74] Wei T.-C., Goldbart P.M. (2003). Geometric measure of entanglement and applications to bipartite and multipartite quantum states. Phys. Rev. A.

[75] Enríquez M., Wintrowicz I., Życzkowski K. (2016). Maximally entangled multipartite states: A brief survey. J. Phys. Conf. Ser..

[76] Olbrich C., Jansen T.L.C., Liebers J., Aghtar M., Strümpfer J., Schulten K., Knoester J., Kleinekathöfer U. (2011). From Atomistic Modeling to Excitation Transfer and Two-Dimensional Spectra of the FMO Light-Harvesting Complex. J. Phys. Chem. B.

[77] Young A.W., Eckner W.J., Schine N., Childs A.M., Kaufman A.M. (2022). Tweezer-programmable 2D quantum walks in a Hubbard-regime lattice. Science.

